# Climate-driven spatial reorganization of cropland nitrogen pollution hotspots toward arid and semi-arid regions

**DOI:** 10.1093/nsr/nwag455

**Published:** 2026-07-24

**Authors:** Jie Qiu, Xing Yan, Di Zhao, Wei Hu, Xuran Li, Zelin Huang, Xiaoyuan Yan, Yongqiu Xia

**Affiliations:** State Key Laboratory of Soil and Sustainable Agriculture, Changshu National Agro‐Ecosystem Observation and Research Station, Institute of Soil Science, Chinese Academy of Sciences, Nanjing 211135, China; College of Resources and Environment, University of Chinese Academy of Sciences, Beijing 100049, China; State Key Laboratory of Soil and Sustainable Agriculture, Changshu National Agro‐Ecosystem Observation and Research Station, Institute of Soil Science, Chinese Academy of Sciences, Nanjing 211135, China; State Key Laboratory of Soil and Sustainable Agriculture, Changshu National Agro‐Ecosystem Observation and Research Station, Institute of Soil Science, Chinese Academy of Sciences, Nanjing 211135, China; College of Resources and Environment, University of Chinese Academy of Sciences, Beijing 100049, China; New Zealand Institute for Bioeconomy Science Limited, Christchurch 8140, New Zealand; Rural Energy and Environment Agency, Ministry of Agriculture and Rural Affairs, Beijing 100125, China; State Key Laboratory of Soil and Sustainable Agriculture, Changshu National Agro‐Ecosystem Observation and Research Station, Institute of Soil Science, Chinese Academy of Sciences, Nanjing 211135, China; State Key Laboratory of Soil and Sustainable Agriculture, Changshu National Agro‐Ecosystem Observation and Research Station, Institute of Soil Science, Chinese Academy of Sciences, Nanjing 211135, China; College of Nanjing, University of Chinese Academy of Sciences, Nanjing 211135, China; State Key Laboratory of Soil and Sustainable Agriculture, Changshu National Agro‐Ecosystem Observation and Research Station, Institute of Soil Science, Chinese Academy of Sciences, Nanjing 211135, China; College of Nanjing, University of Chinese Academy of Sciences, Nanjing 211135, China

**Keywords:** cropland nitrogen runoff, climate change, arid and semi-arid regions, aquatic network removal, water quality management

## Abstract

Cropland nitrogen (N) runoff contributes over 40% of riverine N loads globally, driving widespread aquatic eutrophication. The severity of this pollution is governed by the balance between N runoff and aquatic retention, yet how climate change will redistribute this risk across regions remains unclear. By integrating 1485 field observations into spatially explicit models across China, we show that climate change is creating new N pollution hotspots in arid and semi-arid regions. China’s humid South currently dominates riverine cropland N export (0.31 vs. 0.16 Tg in arid regions). However, under SSP2-4.5 and SSP5-8.5 scenarios, humid regions demonstrate hydrological resilience by 2050, with exports declining by ∼5% due to warming-enhanced aquatic retention. In contrast, cropland N export from dryland-dominated North surges by 18.8%–53.2%, driven by a nonlinear amplification of N runoff. These hydroclimatic shifts necessitate spatially adaptive strategies that prioritize cropland source reduction in the newly emerging arid and semi-arid hotspots.

## Teaser

Climate change is reshaping the geography of cropland nitrogen pollution in China, shifting emerging hotspots from the humid South toward arid and semi-arid northern regions. These findings highlight the need for climate-adaptive nitrogen management that prioritizes source reduction in newly vulnerable regions.

Cropland nitrogen (N) loss is a major source of aquatic pollution globally, contributing over 40% of riverine N loads and driving widespread eutrophication [1,2]. Although N enters aquatic systems via both surface runoff and subsurface leaching, these pathways differ markedly in their transport dynamics [3,4]. Unlike the leaching characterized by diffusion and delayed transport [5,6], surface runoff responds rapidly to rainfall events, delivering large pulses of fertilizer and soil-derived N to water bodies within days [2,7]. These episodic exports often exceed 20 mg N L^−1^ during high flows and dominate acute water quality violations in agricultural basins [8]. Addressing cropland runoff-driven N pulses is therefore critical for managing pollution risks, particularly under a warming climate.

Currently, humid agricultural regions generally dominate cropland N export because abundant rainfall and dense drainage networks promote hydrological connectivity and nutrient transport, whereas arid and semi-arid croplands remain relatively transport-limited. This contrast suggests that humid and dryland systems may respond differently to future hydroclimatic change. Climate change will reshape these pollution risks, with the greatest changes expected in arid and semi-arid regions [9]. Hydrological responses to climate change are projected to diverge sharply across regions under high-emission scenarios (e.g. SSP5-8.5). Annual precipitation is projected to increase by 20%–50% in many arid and semi-arid regions by 2050 (relative to 2019), compared with smaller variability (<15%) in humid regions [10,11] (Fig. S1). Concurrently, warming will accelerate soil N mineralization, enlarging the N pool available for runoff [12]. However, aquatic N removal is also temperature-dependent, and warming enhances in-stream denitrification [13]. These processes interact in complex, often competing ways. Whether they will exacerbate pollution or trigger a spatial reorganization of N export hotspots from humid to arid and semi-arid regions remains uncertain.

Resolving this uncertainty requires capturing nonlinear climate-N coupling across river networks, yet current tools cannot capture these dynamics. Most national-scale assessments rely on export coefficient models with fixed runoff ratios, which obscure nonlinear climate-runoff relationships and ignore temperature-dependent in-stream N retention [14–16]. Although process-based models, such as the Soil and Water Assessment Tool (SWAT) and the DeNitrificationDeComposition (DNDC) model, offer mechanistic realism, their large-scale applications are constrained by substantial data requirements [17,18]. This limitation is particularly acute in data-sparse arid regions, where limited monitoring further compromises model accuracy. Consequently, current modeling frameworks cannot adequately assess how climate-driven shifts will alter the spatial distribution of cropland N pollution risk.

Here, we address this gap by using China as a model system, which spans a pronounced hydroclimatic gradient from the arid North to the humid South. We integrate 1485 field observations into a spatially explicit framework coupling Structural Equation Model (SEM) with a Water Network Framework. This approach enables accurate quantification of the impacts of climate, soil properties, land uses, and river networks on N export across China’s croplands. It requires fewer parameters than traditional models while explicitly representing N runoff and retention within aquatic networks, enabling projection of future risks under Coupled Model Intercomparison Project Phase 6 (CMIP6) climate scenarios. Specifically, we characterize divergent climate-driven N runoff patterns between arid and humid basins, quantify the buffering capacity of river networks, and show how future hydroclimatic shifts will redistribute N export hotspots across China’s agricultural landscape. These insights provide a foundation for climate-adaptive N management strategies tailored to regional vulnerabilities.

## RESULTS AND DISCUSSION

### Divergent climate-driven nitrogen runoff: arid North vs. humid South

**Figure 1.**
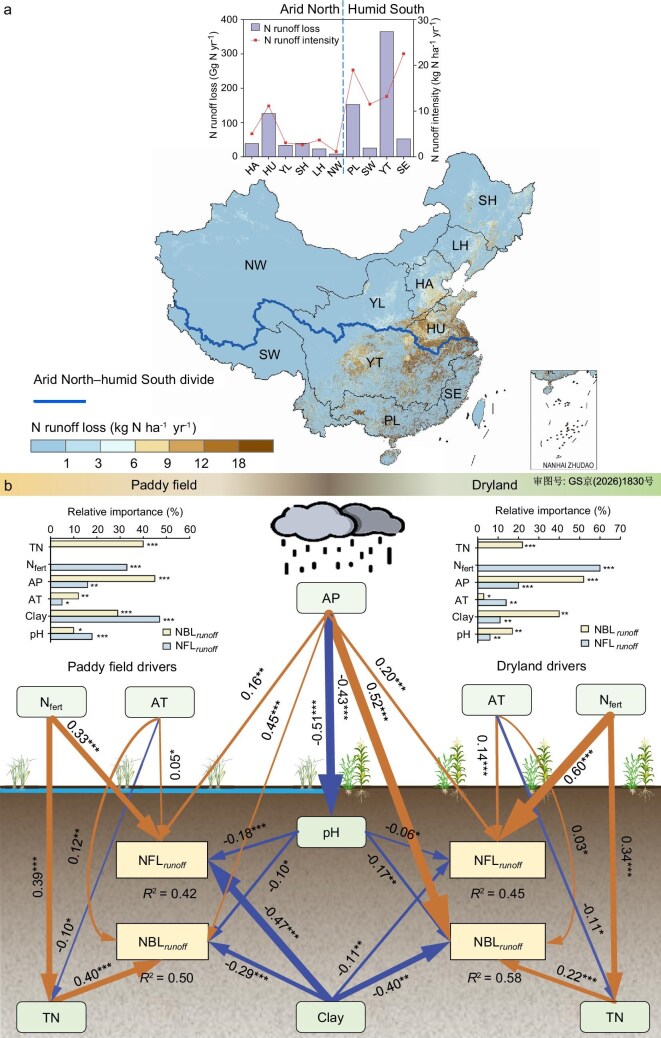
Spatial patterns and climate-driven mechanisms of China’s cropland nitrogen runoff in 2019. (a) Spatial distribution of cropland nitrogen runoff in 2019 (kg N ha^−1^ yr^−1^), with corresponding nitrogen discharge totals for 10 Chinese river basins (Gg N yr^−1^). (b) SEM analysis and relative importance of driving factors on nitrogen background runoff (NBL*_runoff_*) and fertilizer-derived runoff (NFL*_runoff_*) in Paddy fields (*χ*^2^/df = 1.62, *P* = 0.09, *RMSEA* = 0.05, *CFI* = 0.98, *IFI* = 0.98) and Drylands (*χ*^2^/df = 1.72, *P* = 0.06, *RMSEA* = 0.04, *CFI* = 0.98, *IFI* = 0.95). Ten river basins include Hai River Basin (HA), Huai River Basin (HU), Yellow River Basin (YL), Songhua River Basin (SH), Liao River Basin (LH), Northwest River Basin (NW), Pearl River Basin (PL), Southwest River Basin (SW), Yangtze River Basin (YT), and Southeast River Basin (SE). Orange and blue arrows denote positive and negative effects, respectively. The numbers adjacent to the pathway arrows represent the standardized path coefficients, with pathway widths scaled according to their magnitudes. Percentage near endogenous variable indicates the total explanation degree of variation. ***P < 0.001; **P < 0.01; *P < 0.05. NBL*_runoff_*, background N runoff from unfertilized controls; NFL*_runoff_*, fertilizer-derived N runoff calculated as fertilized minus control runoff; TN, soil total N content; N_fert_, N fertilizer application; AP, annual precipitation; AT, annual temperature; Clay, soil clay content; pH, soil pH.

China’s cropland N runoff totaled 0.86 Tg in 2019, with pronounced spatial disparities between the humid southern basins (hereafter ‘humid South’) and the arid and semi-arid northern basins (‘arid North’) (Fig. 1a and Fig. S2). The humid South dominated the national load, contributing 0.59 Tg (68.8%), vs. 0.27 Tg from the arid North. Runoff intensity in the humid South was 14.75 kg N ha^−1^ yr^−1^, nearly three times that in the arid North (4.70 kg N ha^−1^ yr^−1^). Paddy fields accounted for 60.1% of total N runoff in the humid South, whereas drylands contributed 74.2% in the arid North (Fig. S3). Fertilizer application was the primary N source in both regions, while background N runoff, estimated from unfertilized controls, comprised 32.5% of runoff in the humid South and 22.1% in the arid North (Table S1).

SEM revealed contrasting mechanisms through which precipitation and temperature regulate N runoff across China’s hydroclimatic gradient (Fig. 1b). In the arid North drylands, N runoff was restricted by the low transportation capacity and driven by annual precipitation (path coefficient *β* = 0.52, *P* < 0.001). This dependency reflects the high hydrological sensitivity typically found in water-limited agroecosystems. Mechanistically, this coupling reflects the system’s nonlinear response to precipitation variability. To quantify this threshold behavior, we conducted a segmented regression between annual precipitation and N runoff intensity across the arid North. Across 50 repeated random samples, the critical precipitation threshold was consistently identified at ∼457 mm, with a 95% range of 424–476 mm (Fig. S4). Above this threshold, the N runoff response was amplified by about 6-fold, indicating that annual precipitation controls the activation of hydrological connectivity and flow paths in water-limited landscapes [19,20]. Consequently, annual precipitation determines the system’s cumulative mobilization potential, making it a robust driver of export dynamics in these water-limited landscapes. Furthermore, our analysis confirms that increased annual precipitation in the arid North is strongly associated with intensified extreme precipitation events (Fig. S5), reinforcing its role in driving future pulse dynamics. Additionally, temperature exerted a significant, independent effect (path coefficient *β* = 0.14, *P* < 0.001), likely by modulating soil N mineralization rates.

In contrast, N runoff intensity in the humid South was mediated biogeochemically, where the direct influence of hydrological forcing was dampened. While precipitation and temperature collectively explained a comparable portion (57%) of the variance, they operated mainly through indirect, geochemically mediated pathways. Elevated rainfall promoted soil acidification (path coefficient *β* = 0.51, *P* < 0.001), which in turn increased N mobility via lower pH [21] (*β* = −0.18 for NBL*_runoff_; β* = −0.10 for NFL*_runoff_*). Increased temperatures also enhanced N uptake potential by accelerating biological N turnover [22]. Consequently, N runoff in the humid South was less responsive to immediate climate variability, instead exhibiting persistent losses governed by this geochemical mediation.

### Water networks cannot eliminate regional nitrogen export disparities

**Figure 2.**
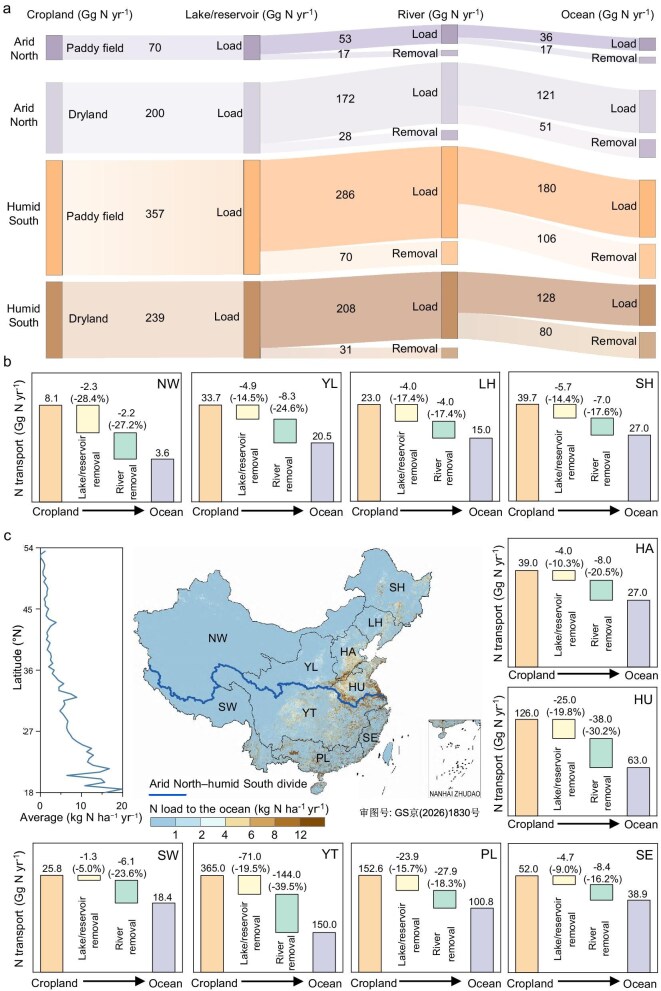
Nitrogen removal and transport across China’s aquatic networks in 2019. (a) Nitrogen transport from paddy fields and drylands to the ocean through water systems. (b) Nitrogen removal and transport through water systems across 10 Chinese river basins. (c) Spatial distribution and latitudinal averages of cropland nitrogen export to the ocean. Values in panels a and b are absolute fluxes; values in panel c are area-normalized intensities and therefore not directly summable to panel-a totals. HA, Hai River Basin; HU, Huai River Basin; YL, Yellow River Basin; SH, Songhua River Basin; LH, Liao River Basin; NW, Northwest River Basin; PL, Pearl River Basin; SW, Southwest River Basin; YT, Yangtze River Basin; SE, Southeast River Basin.

Our analysis reveals that aquatic removal processes were insufficient to offset the stark disparities in terrestrial loading. Nationally, China’s aquatic systems removed 45.9 ± 7.6% of cropland N runoff in 2019. The estimated removal efficiency was 48.3 ± 7.2% in the humid South and 41.6 ± 8.2% in the arid North. This difference in removal efficiency was insufficient to counterbalance the regional imbalance in initial N runoff loads (Fig. 2, Table S2 and Fig. S6). As a result, net N export to the ocean remained 0.31 ± 0.04 Tg in the humid South and 0.16 ± 0.02 Tg in the arid North.

These regional differences in N removal were driven primarily by differences in hydrological network structures and temperature. In the arid North, sparser and largely ephemeral river networks (stream density <0.03 km km^−2^) yielded high relative N removal efficiency during low-flow conditions [12,13] (Fig. S7). However, owing to low temperature, the absolute N removal capacity remained low. In the humid South, dense, perennial drainage networks (>0.05 km km^−2^) combined with high temperature created a high-capacity removal system [23]. This sustained hydrological connectivity and warmer conditions enhanced biogeochemical processing (e.g. denitrification), allowing these systems to remove substantially greater amounts of N (Fig. S7). Thus, while southern humid systems functioned as massive biogeochemical sinks, northern river networks offered minimal hydrological buffering, leaving riverine export directly exposed to terrestrial runoff pulses.

### Climate-driven new nitrogen export hotspots in arid regions

**Figure 3.**
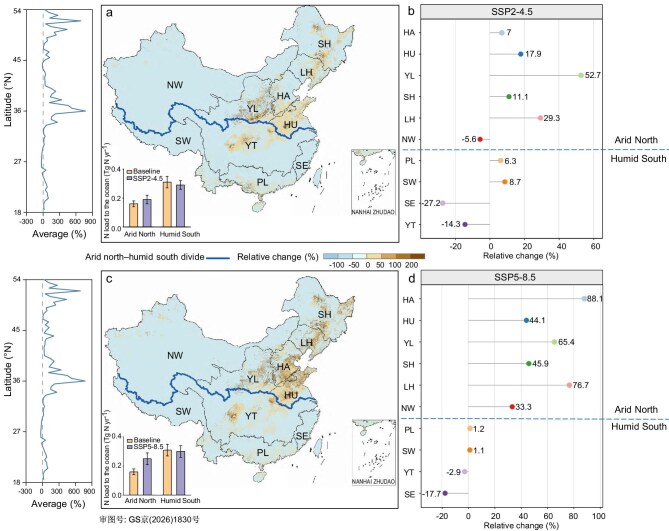
Changes in cropland nitrogen export to the ocean by 2050 under SSP2-4.5 and SSP5-8.5. (a and b) Relative changes in cropland nitrogen export under SSP2-4.5 at national (a) and basin scales (b). (c and d) Relative changes in cropland nitrogen export under SSP5-8.5 at national (c) and basin scales (d). HA, Hai River Basin; HU, Huai River Basin; YL, Yellow River Basin; SH, Songhua River Basin; LH, Liao River Basin; NW, Northwest River Basin; PL, Pearl River Basin; SW, Southwest River Basin; YT, Yangtze River Basin; SE, Southeast River Basin.

Climate change is projected to reshape the geography of cropland N export across China, creating new hotspots in arid North where it was historically less vulnerable (Fig. 3 and Figs S8–S10). Under the moderate-emission scenario (SSP2-4.5), national total cropland runoff-driven N export to the ocean is expected to remain nearly unchanged, increasing slightly from 0.47 ± 0.07 Tg to 0.48 ± 0.07 Tg by 2050. This modest national increase is driven largely by northern arid and semi-arid regions, where projected rises of 21.9% in precipitation and 24.0% in temperature are expected to increase cropland N export by 18.8 ± 6.3% (from 0.16 ± 0.02 Tg to 0.19 ± 0.03 Tg). In contrast, cropland N export in the humid South remains relatively stable. Under the high-emission scenario (SSP5-8.5), national total runoff-driven N export from croplands to the ocean is projected to increase from 0.47 ± 0.07 Tg to 0.55 ± 0.08 Tg by 2050. In this scenario, N export from the arid North rises sharply by 53.2 ± 9.4% (from 0.16 ± 0.02 Tg to 0.25 ± 0.04 Tg), while the humid South shows a marginal decrease (from 0.31 ± 0.04 Tg to 0.30 ± 0.04 Tg) despite projected increases of 14% in precipitation and 10.7% in temperature.

At the basin scale, these contrasts manifest as amplified vulnerability in the North vs. hydrological resilience in the South under the high-emission scenario (SSP5-8.5) (Fig. 3). The Hai River Basin in the arid North shows exceptional sensitivity, where a 60.8% increase in precipitation and 17.7% increase in temperature amplifies cropland N export to the ocean by 88.1%. Similar vulnerabilities are projected for the Yellow and Liao River Basins. Conversely, several southern humid basins demonstrate strong hydrological resilience. The Southeast River Basin, for instance, is projected to reduce cropland runoff-driven N export to the ocean by 17.7% despite projected increases of 5.9% in precipitation and 8.5% in temperature, and the Southwest River Basin remains stable even under projected increases of 34.6% in precipitation and 25.7% in temperature.

Three interacting mechanisms likely underpin these future regional divergences. First, localized decreases in precipitation within some southern humid sub-basins, though masked by overall wetting trends, may reduce pollution in high-loss, rainfall-sensitive areas, offsetting increases elsewhere [21,24,25]. Second, the projected N surge in the arid North likely reflects legacy inorganic N accumulation during prolonged dry periods, with additional contributions from warming-enhanced soil organic N mineralization. Extreme precipitation can rapidly flush this accumulated N pool, generating event-driven pulses that sparse northern drainage networks cannot fully buffer [26,27]. Thus, the projected risk represents pulse release from a long-term N pool, rather than either a purely transient event or a monotonic warming-driven trend. Third, in contrast to the arid North, the humid South’s dense, perennial river networks are expected to sustain high N removal efficiency. This resilience is driven by its hydrological structure and actively enhanced by future warming, which boosts biogeochemical removal (the thermal effect) [13]. Nevertheless, this thermal enhancement should be viewed as bounded, as excessive warming may eventually impair net N removal by altering respiration, oxygen availability, microbial communities, or denitrification pathways. Collectively, the arid North’s emerging vulnerability reflects a synergistic triple interaction of increasing precipitation, elevated N accumulation, and limited hydrological buffering capacity, while the humid South’s resilience is actively enhanced by future warming.

### Toward climate-adaptive nitrogen management: strategies and perspectives

The contrasting patterns of cropland N export identified across China provide a conceptual framework for climate-adaptive pollution management in hydroclimatically diverse agricultural regions worldwide. The high hydrological sensitivity identified in northern China may share similarities with other arid and semi-arid croplands globally, such as Australia’s wheat belt and the North American Great Plains, where accumulated N surplus can be episodically mobilized under intense rainfall. However, regional differences in precipitation regimes, soil properties, agricultural management, and baseline N surplus levels may modulate the magnitude and timing of this vulnerability. These similarities and regional differences underscore the need for locally tailored strategies that explicitly consider climate-N interactions [12,28].

**Figure 4.**
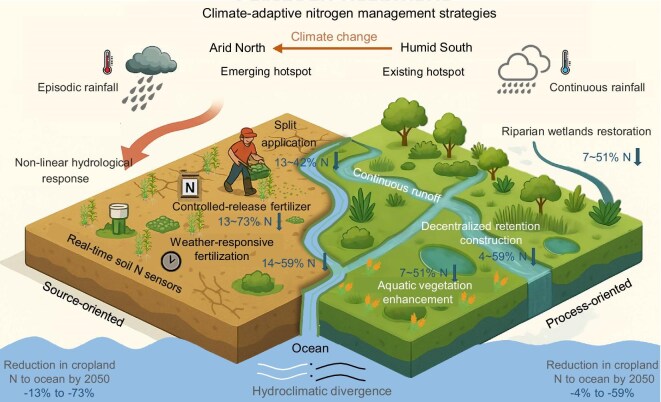
Climate-adaptive nitrogen management strategies.

Our findings highlight the importance of spatially differentiated management strategies tailored to opposing regional vulnerabilities (Fig. 4). In highly vulnerable northern dryland basins (e.g. Hai, Yellow, and Liao Rivers), source-oriented prevention should be prioritized to minimize soil N surpluses available for mobilization. This is critical given the dual threat of precipitation and temperature for N runoff. Meta-analyses and field studies suggest that optimal cropland source control measures targeting excessive N buildup, such as weather-responsive fertilization, controlled-release fertilizers, and split applications, could reduce riverine N export to the ocean by 13%–73% (Fig. 4) [29–31]. These measures primarily act by restricting the accumulation of residual soil N during dry periods, thereby mitigating acute losses during subsequent storm events. In smallholder-dominated systems such as North China, the feasibility of weather-responsive fertilization could be enhanced by digital agriculture platforms, satellite-based precipitation forecasting, and mobile advisory services that translate short-term rainfall predictions into field-level fertilization guidance. Supplementary measures, such as on-farm retention structures and strategically positioned wetlands, are also essential to buffer inevitable runoff pulses and reduce downstream impacts.

In contrast, in humid southern basins characterized by dense and perennial aquatic networks, priorities should be to maintain and enhance in-stream and riparian N removal. Our findings show that the humid South’s resilience is rooted in its hydrological structure and actively enhanced by future warming, which boosts the temperature-dependent biogeochemical removal rates. Therefore, process-based mitigation strategies, including riparian wetland restoration, enhancement of aquatic vegetation, and construction of decentralized retention systems (e.g. ponds and floodplains), can enhance N removal through denitrification, sedimentation, and plant uptake. Model projections suggest these measures could reduce riverine N export to the ocean by 4%–59% (Fig. 4) [32–34], strengthening the humid South’s capacity to cope with future hydrological variability.

Despite these advances, some limitations should be noted. First, we focus exclusively on cropland N runoff, excluding atmospheric deposition, non-cropland sources and groundwater pathways. Groundwater is a major N pathway but often operates with multi-decadal lags [35], whereas surface runoff acts as a fast-response vector to precipitation anomalies. In arid and semi-arid regions, high-intensity precipitation may also enhance episodic recharge, allowing accumulated soil N to move vertically toward groundwater as well as laterally to surface waters. The identified hotspots may therefore reflect multi-pathway N pollution potential, and our runoff-based projections should be viewed as a conservative baseline of total export risk. Second, our Monte Carlo treatment of the temperature correction factor *θ* captures random variability but not systematic regional differences between arid-region and humid-region aquatic systems; if northern streams exhibit elevated *θ* values, our projected northern hotspot magnitudes would be conservative with respect to in-stream attenuation. Third, although using annual precipitation simplifies intra-annual storm dynamics, it robustly captures the non-linear scaling of N export in dryland catchments [1,36]. In these water-limited regions, annual precipitation acts as the primary constraint on total mobilization capacity, effectively integrating the cumulative impact of wet-season pulses. Although this may obscure the timing of individual acute episodes, it reliably constrains the aggregate basin-scale flushing. Future work coupling this framework with sub-daily hydrological models could further resolve the temporal precision of these pulse events.

Our findings signal a critical paradigm shift in water quality management. The geography of cropland runoff-driven N export risk is dynamic, being actively reshaped by climate change. Future rainfall and temperature patterns are likely to overturn existing vulnerability patterns, exposing new hotspots in regions once considered resilient. This underscores the urgent need to move beyond one-size-fits-all management toward regionally adaptive strategies that explicitly integrate climate-N interactions. Although our analysis focuses on China, the identified climate–N interactions may extend to other dryland agroecosystems with comparable hydroclimatic constraints, such as the North American Great Plains and Australia’s wheat belt, while their magnitude and management implications require further region-specific evaluation. A critical next step is to develop next-generation models that couple surface and subsurface hydrology, thereby capturing event-scale storm dynamics and legacy N release that will ultimately govern the emergence of new N pollution hotspots.

## MATERIALS AND METHODS

**Figure 5.**
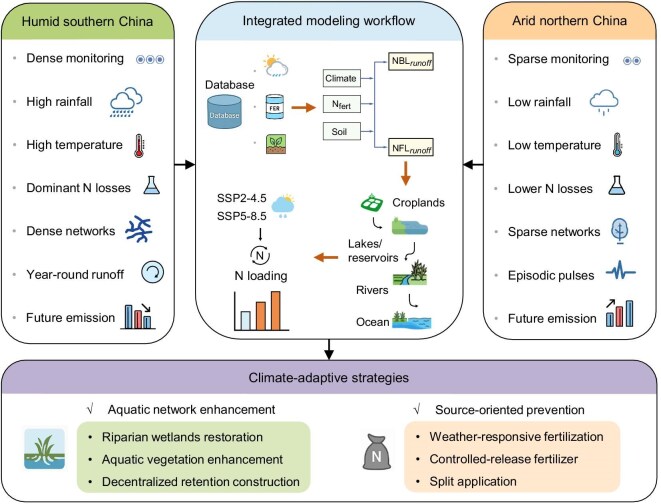
Integrated framework for modeling climate-driven nitrogen runoff, regional contrasts, and climate-adaptive management in China.

Detailed descriptions of the methods (Fig. 5) are available in the Supplementary information.

## Supplementary Material

nwag455_Supplemental_File

## Data Availability

Future climate projections, including annual precipitation and mean annual temperature for 2050 under SSP2-4.5 and SSP5-8.5, were sourced from the National Tibetan Plateau Data Center (http://data.tpdc.ac.cn/) and the Resource and Environment Science and Data Center (https://www.resdc.cn/). Soil properties (clay content, pH, and total N) were obtained from the Soil Science Data Center (https://soilhub.cn/). N fertilization data were sourced from the National Bureau of Statistics (http://www.stats.gov.cn/tjsj/ndsj/). Annual precipitation and temperature data were retrieved from the Resource and Environmental Science and Data Center (https://www.resdc.cn/). Daily precipitation data for observational stations across dryland-dominated northern China were obtained from the National Centers for Environmental Information (NCEI) (https://www.ncei.noaa.gov/). Source data are available at Figshare: https://doi.org/10.6084/m9.figshare.30608423.
