# Supplementary material for: Climate-driven spatial reorganization of cropland nitrogen pollution hotspots toward arid and semi-arid regions
Source: Natl Sci Rev. 2026 Jul 24;13(15):nwag455. doi: 10.1093/nsr/nwag455 (PMC13471033; doi:10.1093/nsr/nwag455)
Supplement: Supplementary file 1 [file nwag455_supplemental_file.docx]

**Supplementary Information for**

Climate-driven spatial reorganization of cropland nitrogen pollution hotspots toward arid and semi-arid regions

Jie Qiu^1,2^, Xing Yan^1^, Di Zhao^1,2^, Wei Hu^3^, Xuran Li^4^, Zelin Huang^1^, Xiaoyuan Yan^1,5^, Yongqiu Xia^1,5*^

^1^ *State Key Laboratory of Soil and Sustainable Agriculture,* *Changshu National Agro-Ecosystem Observation and Research Station, Institute of Soil Science, Chinese Academy of Sciences, Nanjing, 211135, China*

^2^ *University of Chinese Academy of Sciences, Beijing, 100049, China*

^3^ *New Zealand Institute for Bioeconomy Science Limited, Private Bag 4704, Christchurch 8140, New Zealand*

^4^ *Rural Energy and Environment Agency, Ministry of Agriculture and Rural Affairs, Beijing, 100125, China*

^5^ *University of Chinese Academy of Sciences, Nanjing, 211135, China*

^*^ Correspondence: [yqxia@issas.ac.cn](mailto:yqxia@issas.ac.cn) (Y. Xia)

**This PDF file includes:**

Supplementary Method, Tables S1–S6, Figs S1–S15, and References 1–259.

**Supplementary Methods**

To comprehensively assess the impact of precipitation and temperature on cropland N runoff and its fate in aquatic systems, a four-step methodological framework was employed (Fig. 5). First, we compiled an extensive dataset of field-scale N runoff observations across China through a systematic literature review, supplemented by quality-control procedures. Second, we developed a Structural Equation Model (SEM) based on established biogeochemical theory to quantify the mechanistic pathways through which precipitation, temperature, soil properties, and fertilization practices jointly regulate background and fertilizer-driven N runoff losses [1]. Third, we spatially extrapolated cropland N runoff losses nationwide and simulated their transport and retention along waterways using the Water Network Framework (WNF) with independent parameter calibration and validation [2-5]. Finally, we conducted scenario-based simulations to estimate changes in N export under future precipitation and temperature projections for 2050. Detailed procedures for each step are described below.

**Study area**

To explicitly disentangle the distinct N mobilization and retention dynamics across China’s croplands, we focused on ten major agricultural basins that collectively represent the country's primary production regions. The basin delineation followed the major river-basin zoning framework used in previous national-scale inland-water N assessments [6] (Fig. S11). The study area was further partitioned into two contrasting hydro-climatic zones following the conventional 800-mm precipitation threshold (Qinling–Huaihe Line) [7].

The dryland-dominated arid North (including the Hai, Huai, Yellow, Northwest, Liao, and Songhua basins) is characterized by water-limited conditions where precipitation acts as a primary constraint on N transport. In contrast, the humid South (including the Yangtze, Pearl, Southeast, and Southwest basins) represents water-abundant systems where biological processing plays a more dominant role. Basin-level cropland area and cropland percentage were calculated by overlaying the 1-km cropland map with basin boundaries, with paddy fields and drylands summarized separately (Table S3). This zonal classification provides the structural basis for our region-specific modeling of N export risks under changing climate.

**Structural Equation Model (SEM)**

To elucidate the direct and indirect effects of climate change on cropland N runoff, we employed SEM to quantify the relationships among precipitation, temperature, soil properties, and N runoff intensity. The SEM was selected over traditional regression because of its unique ability to test complex, theory-driven causal pathways, distinguishing direct hydro-biogeochemical effects of precipitation and temperature from indirect effects mediated by soil properties. SEM enables the testing of hypothesized causal pathways (Fig. S12) informed by established biogeochemical theories: (i) Precipitation directly drives N runoff through hydrological mobilization. For this pathway, we selected annual precipitation as the primary driver variable. We chose annual precipitation because, in the arid North, annual precipitation totals are strongly and positively correlated with the frequency and intensity of extreme rainfall events (Fig. S5), which are the dominant mechanism for generating surface runoff pulses. Annual precipitation thus serves as a robust statistical proxy for the precipitation-limited N export capacity in these arid and semi-arid systems; (ii) temperature directly affects N runoff by regulating the soil N mineralization rate; (iii) precipitation and temperature indirectly influence N runoff through their effects on soil acidification and the soil N pool, which subsequently enhances N mobility; and (iv) soil texture (clay content) modulates N retention capacity and runoff response [1,8,9].

The SEM analysis was based on 1,485 field observations compiled from a peer-reviewed literature (Web of Science, Google Scholar, and CNKI, cutoff = 2025). Search terms included combinations of “nitrogen loss”, “nitrogen fertilization”, “cropland”, and “surface runoff”. Inclusion criteria required studies to (1) be field-based agricultural systems, excluding pot or shelter experiments and modeling studies; (2) report annual N runoff losses across full crop cycles; and (3) include unfertilized controls. Data were standardized (kg N ha^-1^ yr^-1^) and quality-checked to remove implausible outliers. The final dataset included 676 observations from paddy fields and 809 from drylands from 191 publications (Fig. S11 and Table S4).

The data captured a broad climatic gradient. Observations from the arid North (n = 304) spanned 35–54°N with mean annual precipitation of 200–800 mm, concentrated in the North China Plain (59%) and Northeast Plain (27%). Observations from the humid South (n = 1,181) covered 18–35°N with 800–1800 mm precipitation, primarily from rice-intensive regions (Yangtze River Valley: 77%, South China: 20%). The temporal distribution spanned 1999–2025, with peak sampling during 2010–2020 (64% of observations). The compiled dataset included four categories: (1) bibliographic metadata (authors, publication year, experimental period); (2) site characteristics (location, precipitation, temperature, soil properties, cropping systems); (3) management practices (fertilizer rates, types); and (4) N runoff losses, partitioned into background and fertilizer-derived components. Background N runoff was defined as N runoff loss from unfertilized control plots, while fertilizer-derived N runoff was calculated as the difference between total N runoff in fertilized treatments and N runoff in paired unfertilized controls. When site-specific precipitation or soil data were unavailable, values were supplemented from the Chinese Soil Database and Statistical Yearbooks. Data extraction was conducted from published tables and texts, and figures were digitized using GetData Graph Digitizer (v2.26) where necessary.

All variables were standardized prior to modeling. The SEM was implemented in AMOS 25.0 (IBM SPSS, New York, USA), with maximum likelihood estimation. Paths with non-significant coefficients (*P* > 0.05) were iteratively removed to improve model parsimony. Model fit was assessed using chi-square to degrees of freedom ratio (*χ*^2^/df < 2 with *P* > 0.05), the Root Mean Square Error of Approximation (*RMSEA* ≤ 0.05), Comparative Fit Index (*CFI* ≥ 0.95), and Incremental Fit Index (*IFI* ≥ 0.95). To ensure robust generalizability, the dataset was randomly partitioned into calibration (80%) and validation (20%) subsets, with final performance evaluation conducted on the complete observational series (Fig. S13). The SEM results provided mechanistic insights into regional N runoff pathways, informing subsequent scenario analyses.

**Spatial modeling and validation**

Spatial modeling began by estimating sub-basin N runoff loads from region-specific SEM relationships. The SEM analysis revealed distinct climate-N runoff linkages for water-limited and humid basins, quantifying the statistical relationships between N runoff intensity (kg N ha^-1^ yr^-1^) and key drivers including annual precipitation, temperature, soil pH, and clay content. These relationships were applied to gridded datasets of precipitation, temperature, soil properties, and land use (LUCC) to spatially extrapolate N runoff intensities across China's agricultural regions.

The SEM-derived N runoff intensities (in kg N ha^-1^ yr^-1^) were then multiplied by the gridded cropland area data (differentiated by paddy fields and drylands) to generate a high-resolution national map of initial N runoff loads. This spatially explicit map of terrestrial N inputs served as the direct upstream source term for the subsequent aquatic transport simulations using the Water Network Framework (WNF) [2-5]. We justify upscaling the field-scale SEM relationships to the national 1-km grid based on a two-stage source–transport coupling strategy. First, the SEM quantifies the mobilization potential at the grid scale (1 × 1 km), capturing how local climate and soil conditions determine the intensity of N pulses released from the soil matrix. We assume these field-derived relationships are representative of the dominant runoff generation mechanisms within each grid cell. Second, the transition from 'edge-of-field' runoff to downstream export was not represented by a uniform delivery ratio. Instead, each grid-level N source was dynamically routed along its hydrologically connected downstream pathway in the WNF. During routing, the WNF calculates spatially distributed retention based on hydrological connectivity, flow path length, and waterbody-specific attenuation processes. Furthermore, the decay coefficients ($k_{w(T)}$) in the WNF were calibrated against basin-outlet observations, implicitly accounting for terrestrial transmission losses and scaling effects within the coupled framework.

N transport and retention through aquatic networks were simulated using the WNF, a hydrological-nutrient routing model designed to trace N fluxes from terrestrial runoff sources through river systems to oceanic outlets. The WNF was selected for its balance between computational efficiency and mechanistic representation of in-stream nutrient retention, crucial for data-sparse regions such as dryland-dominated northern China. The WNF explicitly represents riverine and lacustrine retention processes, incorporating spatial variations in hydrological connectivity, channel morphology, and waterbody residence times. The WNF model operates on nested 1-km grid cells, tracing flow accumulation pathways from each N source using D8 flow direction algorithms. For each source cell, the model tracks flow paths and quantifies the cumulative lakes/reservoirs area traveled and cumulative length of rivers along the flow direction, applying retention coefficients ($k_{w(T)}$) representing first-order decay processes. These cumulative metrics are then combined with water body-specific removal coefficients to calculate nutrient retention and transport (Fig. S14). N loading ($NL_{w}^{i}$) and removal ($NR_{w}^{i}$) from grid *i* in water body type *w* were estimated as:

$\begin{aligned} NL_{w}^{i}=N_{w}^{i}\times\prod_{j=1}^{n} R_{lj}\times\prod_{k=1}^{m} R_{ak}\#(1) \end{aligned}$

$\begin{aligned} NR_{w}^{i}=\sum_{i=1}^{n} {(N}_{w}^{i})\times\left( 1-R_{w} \right)\left( \mathrm{when}w=1 \right)\#(2) \end{aligned}$

$\begin{aligned} NR_{w}^{i}={[\frac{R_{w-1}}{1-R_{w-1}}C}_{ws}^{w-1}+\sum_{j=1}^{m} {(N}_{w}^{j})]\times\left( 1-R_{w} \right)\left( \mathrm{when}w>1 \right)\#(3) \end{aligned}$

where $N_{w}^{i}$ refers to the N runoff from grid cell 𝑖; $R_{a}\mathrm{and}$ $R_{l}$represent discharge fractions for the areal and linear water body, respectively; $R_{w}$ and $R_{w-1}$are the N load fractions of the *w*th water and the upstream water of the *w*th water (*w*-1); ${1-R}_{w}$ is the fraction of the nutrients removed by the *w*th water; $C_{ws}^{w-1}$ is the nutrients removed by the upstream water of the *w*th water within the subwatershed (*s*). If the water is the first order or the outermost of the watershed, all sources loading ($N_{w}^{i}$) in the subwatershed will be equally removed. If the water is not in the outermost part of the subwatershed, the discharge from the water upstream (${\frac{R_{w-1}}{1-R_{w-1}}C}_{ws}^{w-1}$) and other sources ($N_{w}^{j}$) in the subwatershed will be equally removed, and so on.

The release factor of nutrients during water transport ($R_{a}$ and $R_{l}$) is determined by a removal rate ($k_{w(T)}$) assigned to each unit of travel time (*t*), expressed as follows:

$\begin{aligned} R=\exp\left( -k_{w(T)}t \right)\#(4) \end{aligned}$

To incorporate the strong influence of temperature on in-stream biogeochemical processing, the removal rate $k_{w(T)}$ was not treated as a static constant. Instead, it was dynamically calculated based on the mean annual air temperature (*T*) of each grid cell. This calculation was a two-step process: First, we established baseline removal rates at a 20°C reference temperature ($k_{w(20)}$) using values derived from empirical studies, considering variables such as water type, size, and location. For lakes/reservoirs, we adopted daily removal rates reported by Shen et al. [10] for 417 Chinese waterbodies, which were annualized and converted by water surface area. For rivers, we applied reach-specific removal coefficients from Keller et al. [11], with the first to fourth river types in our study corresponding to very large, large, medium, and small rivers, respectively, based on 95% confidence intervals of size-specific means (Table S5). Second, the final, temperature-specific removal rate ($k_{w(T)}$) for each grid cell was calculated by adjusting this baseline rate using the standard $Q_{10}$ approach, which is expressed as:

$$\begin{aligned} k_{w\left( T \right)}=k_{w\left( 20 \right)}\cdot\theta^{\left( T-20 \right)}\#\left( 5 \right) \end{aligned}$$

where $k_{w(T)}$ is the adjusted removal rate for a given temperature *T*. $k_{w(20)}$ is the baseline removal rate at 20°C (from Shen et al. [10] and Keller et al. [11]). $\theta$ is the temperature correction factor, representing the sensitivity of the removal processes (primarily denitrification). Based on established literature, this value was set to 1.047 [12].

Travel times for areal water bodies ($t_{a}$) correlate with the surface area ($A_{a}$), while those for linear water bodies ($t_{l}$) relate to the hydraulic path length${(l}_{i})$and the average slope of the path [13,14].

$\begin{aligned} t_{a}=\beta_{1}{{(A}_{a})}^{\beta_{2}}=\beta_{1}{(\sum_{j=1}^{m} A_{aj})}^{\beta_{2}}\#(6) \end{aligned}$

$\begin{aligned} t_{l}=\frac{L}{v}=\frac{1}{n}\sum_{j=1}^{n} \frac{l_{i,j}}{{R_{i,j}}^{\frac{2}{3}}\sqrt{s_{i,j}}}\#\#\left( 7 \right) \end{aligned}$

in which *n* is the number of liner water bodies localized along the hydraulic path *i*; $A_{aj}$ is the area of nutrients passing through water *j*; $l_{i,j}$, $R_{i,j}$, and $s_{i,j}$are the length, hydraulic radius, and average slope of *i*th source localized to water *j* along the hydraulic path, respectively. The coefficients $\beta_{1}$(equal to 1.5) and $\beta_{2}$(equal to 0.23) were derived from a comprehensive meta-analysis of various areal waters worldwide.

All spatial analyses were conducted on a gridded basis using ArcGIS 10.5, incorporating digital elevation maps (DEM) (<https://www.resdc.cn/Default.aspx>) and water system datasets (Fig. S15). A 60-meter DEM burning process was conducted to enhance the representation of small waterbodies. River networks were classified into four stream orders (1st–3rd, 4th, 5th, 6th) to capture removal capacity variation.

The framework's spatial explicitness enables comprehensive assessment of N loads from agricultural sources through aquatic systems to coastal outlets, providing critical insights for water quality management.

**Scenario analysis**

To isolate the independent impacts of future climate change on N runoff, our scenario analysis assumes all non-climatic anthropogenic drivers are held constant at their 2019 levels. This includes land use distribution, cropping systems, and N fertilization rates. With this assumption, we designed scenario analyses based on two CMIP6 climate trajectories for 2050: SSP2-4.5 (medium emissions) and SSP5-8.5 (high emissions). These two scenarios were chosen to represent moderate and extreme climate-change pathways and to capture a wide range of plausible futures for arid and semi-arid regions. This allows for a robust 'stress test' of the water-limited agro-ecosystem's vulnerability to coupled hydro-climatic shifts (both precipitation and temperature), which is the central hypothesis of this study. SSP2-4.5 reflects a stabilization trajectory with intermediate socio-economic development and mitigation efforts, while SSP5-8.5 represents a high-emission, fossil-fuel-intensive development pathway. Future projections of precipitation and annual mean temperature (1-km monthly projections) for 2050 under both SSPs were sourced from the National Tibetan Plateau Data Center and the Resource and Environment Science and Data Center, respectively.

Our scenario analysis followed a three-step workflow. First, we obtained gridded future precipitation and temperature data for 2050 under the SSP2-4.5 and SSP5-8.5 scenarios. Second, these future data were used as inputs for the region-specific, climate-sensitive SEM-derived statistical relationships to generate high-resolution maps of projected N runoff loads across China's croplands. Finally, these projected N runoff load maps were fed into the WNF model to simulate the downstream transport, retention, and ultimate export of N to the ocean under each climate scenario. Crucially, the future temperature grids were also used to dynamically calculate the temperature-dependent removal coefficient ($k_{w(T)}$) for each grid cell and scenario, allowing the model to simulate N transport and retention under future thermal regimes. This analytical framework allowed us to quantify the integrated impacts of future changes in both precipitation (hydrological driver) and temperature (biogeochemical driver) on N runoff and aquatic transport, providing clear mechanistic insights into the isolated effects of projected precipitation and temperature change.

**Management scenarios for climate-adaptive nitrogen reduction**

To parameterize N reduction scenarios, we conducted a systematic literature review (search terms: ["nitrogen runoff", "mitigation", "China", "paddy", "cropland"], database: Web of Science & CNKI, period: 2000–2025) and meta-analysis to synthesize reported reduction efficiencies of agricultural and hydro-biogeochemical interventions across China's diverse agro-climatic regions. Studies were included if they were field-scale experiments conducted in China lasting at least one full cropping cycle. This process yielded 49 relevant publications, from which the median efficiency for each management practice was derived. A comprehensive inventory of included studies is detailed in Table S6. To align with our hydro-climatic framework, relevant publications were stratified by region into the arid North (Hai, Yellow, and Liao) and the humid South (Yangtze, Pearl, Southeast, and Southwest), as well as by intervention typology. This classification explicitly separates source-oriented controls from process-based removal strategies. Reduction efficiencies were standardized as percentage reductions in N runoff loads relative to baseline conditions.

To construct realistic management scenarios, we identified three widely adopted and consistently reported measures for each region. For the arid North’s basins, we selected weather-responsive fertilization, controlled-release fertilizers, and split applications [15]. These source-oriented measures are recognized as most effective for minimizing soil N accumulation, directly countering the pulse-driven runoff risk identified in our analysis. For the humid South’s basins, we chose process-oriented interventions, riparian wetland restoration, aquatic vegetation management, and decentralized retention ponds, to capitalize on the region’s high N-removal potential within dense, perennial aquatic networks [16-17].

To robustly account for the inherent uncertainty in management effectiveness, we fully integrated the efficiency ranges derived from our meta-analysis into the existing Monte Carlo uncertainty framework. Rather than relying on deterministic coefficients, we parameterized the reduction efficiencies as probability density functions (PDFs) specific to each hydro-climatic regime: 13–73% for source-control measures in the arid North and 4–59% for process-oriented strategies in the humid South. During the 10,000 Monte Carlo iterations, a specific reduction efficiency value was randomly sampled from these region-specific PDFs for each simulation run. This sampled coefficient was then applied mechanistically via our two distinct pathways. For the arid North (Source-Oriented), the sampled efficiency value was used to proportionally decrease the initial 'runoff N loads' generated from each cropland grid cell. In contrast, for the humid South (Process-Oriented), the sampled efficiency value was used to proportionally increase the baseline reference removal coefficient ($k_{w(20)}$) within the WNF model's temperature-dependent removal function.

This probabilistic approach ensures that our final projections for N reduction (Fig. 4) fully propagate the known, field-level variability of management interventions, yielding a far more robust and realistic assessment of mitigation potential than single-point estimates. All simulations were conducted holding baseline climatic and land-use drivers constant to isolate the specific effect of the interventions.

**Uncertainty analysis**

To quantify the propagation of uncertainty in projected N exports, we conducted Monte Carlo simulations (10,000 iterations) incorporating variability from key sources throughout the modeling framework. Uncertainties in input parameters were defined based on observed variability, literature ranges, and ensemble spreads. Specifically, uncertainties in the SEM parameters were defined by the 95% confidence intervals of the standardized path coefficients, resulting in a variation of ± 7–38%. Spatial extrapolation uncertainties were accounted for by applying ± 25–37% variation to N runoff estimates in data-sparse regions, particularly affecting the SEM projections for arid North China where observational data was less dense (n = 304) compared to the humid South (n = 1,181).

Within the WNF, retention coefficient uncertainty was defined by applying variation to the baseline $k_{w(20)}$ parameters (± 4–31%) and the temperature correction factor $\theta$ (± 5–18%) in the $k_{w(T)}$ function, representing the interquartile range derived from empirical, national-scale monitoring datasets [10,11]. The uncertainty range for *θ* was included to account for potential regional and habitat-level variability in the temperature sensitivity of aquatic N removal and was propagated through the Monte Carlo simulations. We note, however, that this Monte Carlo treatment captures random variability around a central 𝜃 value but does not represent systematic regional differences. Empirical evidence suggests 𝜃 may be elevated in arid-region streams (~1.06–1.10) where carbon limitation amplifies temperature sensitivity, relative to humid-region rivers (~1.04–1.06) [10,18]; region-specific 𝜃 measurements would help further constrain this aspect of the model in future work. Climate projection uncertainty was captured through the interannual spread (± 6–9%) of the CMIP6 ensemble for 2046–2055, representing inherent variability in future precipitation and temperature. In addition, N source loss variability (± 9–15%) was incorporated to account for interannual fluctuations in runoff under climate change. These uncertainties were propagated through the modeling chain, from N runoff mobilization to aquatic transport, using established runoff–retention pathways.

Variance-based sensitivity analysis identified precipitation variability as the dominant driver of total uncertainty, contributing 34–38% of projected variance, followed by fertilization rates (24–28%) and soil properties (17–22%). Aquatic retention parameters were secondary but non-negligible, with the baseline aquatic removal coefficient $k_{w(20)}$ and the temperature correction factor *θ* contributing 9–13% and 4–7%, respectively. Residual interaction terms and unexplained variability accounted for the remaining 1–3%. These results indicate that projected N export uncertainty was controlled primarily by source-side N mobilization, especially precipitation-driven runoff generation and fertilizer inputs, whereas aquatic removal parameters regulated downstream retention.

Distinct regional differences in uncertainty were also observed. Water-limited basins exhibited higher uncertainty (coefficient of variation, CV = 38–44%) due to threshold-driven nonlinearity in hydrological and temperature responses, while humid basins showed lower uncertainty (CV = 25–30%), reflecting more stable and predictable runoff dynamics. To further test the fixed-fertilizer assumption, we conducted a bounding sensitivity analysis by perturbing the 2019 gridded fertilizer inputs by ±20% under future climate scenarios while preserving their observed spatial distribution. The ±20% range was selected to bracket the central tendency of SSP-based projections for China's N fertilizer use by 2050, drawn from integrated assessment model (IAM) ensembles. Specifically, Mogollón et al. [19] report that under SSP2 the inter-model spread of China's 2050 N fertilizer inputs falls within approximately ±15–25% of contemporary levels, and Bodirsky et al. [20] provide consistent estimates across multiple IAM frameworks. We acknowledge that the upper tail of SSP5 (agricultural intensification) and the lower tail of SSP1 (sustainability pathways combined with China's national fertilizer-reduction policies) may extend beyond this range. The present test is therefore best interpreted as a mid-range bounding of plausible fertilizer trajectories rather than the full SSP envelope, consistent with our analytical focus on isolating hydroclimatic drivers rather than co-varying management futures. This perturbation modulated the magnitude of projected N export but did not eliminate the hydroclimatic vulnerability identified in the arid North, supporting the use of fixed-management scenarios as climate-forcing baselines. These findings underscore the need for region-specific risk assessments in developing adaptive N management strategies.

Furthermore, for the management scenario simulations, uncertainty in mitigation efficiencies (e.g., 13–73% for the arid North, 4–59% for the humid South) was incorporated by sampling from the region-specific probability density functions derived from our meta-analysis, as described above.

**Table S1. Nitrogen export to the ocean from background and fertilizer sources across 10 Chinese river basins in 2019 under different bio-removal coefficients (Gg).**

| **Basin** | ***k*_min_** | | ***k*_max_** | |  |
| --- | --- | --- | --- | --- | --- |
|  | **Background** | **Fertilizer** | **Background** | **Fertilizer** | |
| **HA** | 6.7 | 23.1 | 5.3 | 18.9 | |
| **HU** | 14.3 | 61.8 | 9.7 | 40.2 | |
| **PL** | 5.1 | 17.7 | 3.9 | 14.3 | |
| **SW** | 8.7 | 20.4 | 7.3 | 17.6 | |
| **YL** | 4.4 | 12.0 | 3.6 | 10.0 | |
| **SE** | 0.7 | 3.4 | 0.5 | 2.6 | |
| **LH** | 38.1 | 71.8 | 31.5 | 60.2 | |
| **NW** | 7.2 | 13.5 | 5.6 | 10.5 | |
| **YT** | 56.1 | 121.8 | 37.9 | 84.2 | |
| **SH** | 14.1 | 28.3 | 11.7 | 23.7 | |

Note: The minimum biological removal coefficients (*k*_min_) for lakes and reservoirs were 0.63% per hectare, and for the first-fourth type rivers, they were 0.13, 0.19, 0.33, and 0.39% per kilometer. The maximum biological removal coefficients (*k*_max_) for lakes and reservoirs was 0.71% per hectare and for first-fourth type rivers were 0.23, 0.83, 0.61, and 0.74% per kilometer. SW, Southwest River Basin; SH, Songhua River Basin; YL, Yellow River Basin; YT, Yangtze River Basin; NW, Northwest River Basin; SE, Southeast River Basin; LH, Liao River Basin; PL, Pearl River Basin; HA, Hai River Basin; HU, Huai River Basin.

**Table S2. Removal percentage (%) from cropland nitrogen runoff in lakes and reservoirs, and rivers in 10 Chinese river basins under different bio-removal coefficients.**

| **Basin** | ***k*_min_** | | ***k*_max_** | |  |
| --- | --- | --- | --- | --- | --- |
|  | **Lake or reservoir** | **River** | **Lake or reservoir** | **River** | |
| **HA** | 9.9 | 12.4 | 10.3 | 26.7 | |
| **HU** | 20.0 | 19.2 | 20.7 | 38.9 | |
| **PL** | 15.5 | 12.8 | 16.2 | 23.8 | |
| **SW** | 4.6 | 14.6 | 4.8 | 32.1 | |
| **YL** | 11.9 | 20.2 | 12.4 | 34.5 | |
| **SE** | 9.4 | 9.9 | 9.9 | 22.8 | |
| **LH** | 15.0 | 13.5 | 15.5 | 24.7 | |
| **NW** | 27.1 | 18.9 | 28.3 | 29.7 | |
| **YT** | 18.7 | 32.4 | 19.5 | 47.0 | |
| **SH** | 14.1 | 13.3 | 14.6 | 23.2 | |
| **National** | 16.3 | 22.2 | 17.0 | 36.5 | |

Note: The minimum biological removal coefficients (*k*_min_) for lakes and reservoirs were 0.63% per hectare, and for the first-fourth type rivers, they were 0.13, 0.19, 0.33, and 0.39% per kilometer. The maximum biological removal coefficients (*k*_max_) for lakes and reservoirs was 0.71% per hectare and for first-fourth type rivers were 0.23, 0.83, 0.61, and 0.74% per kilometer. SW, Southwest River Basin; SH, Songhua River Basin; YL, Yellow River Basin; YT, Yangtze River Basin; NW, Northwest River Basin; SE, Southeast River Basin; LH, Liao River Basin; PL, Pearl River Basin; HA, Hai River Basin; HU, Huai River Basin.

**Table S3. Basin area and cropland composition across the 10 Chinese river basins.**

| **Basin** | **Total area (Mha)** | **Cropland area (Mha)** | **Cropland percentage (%)** | **Paddy field area (Mha)** | **Dryland area (Mha)** |
| --- | --- | --- | --- | --- | --- |
| **HA** | 32.11 | 14.62 | 45.52 | 0.18 | 14.43 |
| **HU** | 32.99 | 21.80 | 66.08 | 4.93 | 16.86 |
| **PL** | 57.50 | 13.32 | 23.16 | 6.30 | 7.02 |
| **SW** | 86.18 | 3.53 | 4.09 | 0.68 | 2.85 |
| **YL** | 81.76 | 19.85 | 24.28 | 0.61 | 19.25 |
| **SE** | 23.90 | 3.71 | 15.50 | 2.82 | 0.89 |
| **LH** | 31.72 | 11.19 | 35.29 | 0.97 | 10.22 |
| **NW** | 338.13 | 12.89 | 3.81 | 0.01 | 12.88 |
| **YT** | 182.20 | 47.72 | 26.19 | 23.42 | 24.30 |
| **SH** | 93.50 | 26.96 | 28.84 | 5.16 | 21.81 |

**Table S4. List of references used for constructing nitrogen runoff emission database.**

| **Study site** | **Crop type** | **Data volume** | **Reference** |
| --- | --- | --- | --- |
| Hubei Province | Cotton | 1 | Bu et al., 2009 [21] |
| Shanghai | Rice | 12 | Cao et al., 2016 [22] |
| Zhejiang Province | Sweet potato | 3 | Chen et al., 2023 [23] |
| Hunan Province | Maize | 12 | Chen et al., 2024 [24] |
| Jiangsu Province | Rice, wheat | 16 | Chen et al., 2016 [25] |
| Liaoning Province | Rice | 5 | Chen et al., 2012 [26] |
| Tianjin | Rice | 1 | Chen et al., 2011 [27] |
| Zhejiang Province | Rice | 4 | Chen et al., 2021 [28] |
| Sichuan Province | Maize, wheat | 5 | Chen et al., 2014 [29] |
| Yunnan Province | Other | 1 | Chu et al., 2010 [30] |
| Liaoning Province | Maize | 3 | Cong and Li, 2021 [31] |
| Sichuan Province | Rice | 5 | Deng et al., 2022 [32] |
| Shanxi Province | Maize, wheat, other | 3 | Dong, 2016 [33] |
| Jiangsu Province | Other | 1 | Duan et al., 2007 [34] |
| Hunan Province | Rice | 9 | Duan, 2018 [35] |
| Hunan Province | Rice | 2 | Duan et al., 2017 [36] |
| Hunan Province | Maize, rape seed | 9 | Duan et al., 2013 [37] |
| Guizhou Province | Maize, rape seed | 18 | Fan et al., 2014 [38] |
| Jiangsu Province | Maize, rice, wheat | 32 | Fan, 2016 [39] |
| Jiangsu Province | Rice | 2 | Fan et al., 2015 [40] |
| Shaanxi Province | Other | 3 | Fu et al., 2004 [41] |
| Hunan Province | Maize | 4 | Gao et al., 2015 [42] |
| Hunan Province | Maize | 1 | Gao et al., 2014 [43] |
| Chongqing | Maize, sweet potato, rice, other | 4 | Gao et al., 2012 [44] |
| Anhui Province | Rape seed | 1 | Gong et al., 2019 [45] |
| Jiangsu Province | Rice | 2 | Gu et al., 2009 [46] |
| Heilongjiang Province | Rice | 6 | Gu et al., 2021 [47] |
| Heilongjiang Province | Rice | 5 | Gu et al., 2017 [48] |
| Anhui Province | Wheat | 7 | Guo et al., 2022 [49] |
| Yunnan Province | Other | 6 | Guo et al., 2023 [50] |
| Jiangsu Province | Vegetable | 2 | Guo et al., 2018 [51] |
| Jiangsu Province | Rice | 1 | Guo et al., 2011 [52] |
| Jiangsu Province | Wheat | 1 | Guo et al., 2011 [53] |
| Chongqing | Maize, wheat | 4 | He et al., 2012 [54] |
| Guangxi Province | Maize, peanut, other | 6 | He et al., 2014 [55] |
| Hunan Province | Maize | 4 | He, 2017 [56] |
| Anhui Province | Rape seed | 6 | Hou et al., 2019 [57] |
| Fujian Province | Rice | 3 | Huang et al., 2013 [58] |
| Hunan Province | Rice | 7 | Huang, 2020 [59] |
| Shanghai | Rice, wheat, vegetable | 6 | Huang et al., 2005 [60] |
| Hunan Province | Maize | 1 | Huang et al., 2010 [61] |
| Shanghai | Vegetable | 7 | Huang et al., 2007 [62] |
| Chongqing | Maize, wheat | 3 | Huo et al., 2013 [63] |
| Hunan Province | Rice | 3 | Ji et al., 2006 [64] |
| Hunan Province | Rice | 5 | Ji et al., 2007 [65] |
| Hunan Province | Rice | 3 | Ji et al., 2007 [66] |
| Yunnan Province | Rice | 14 | Jiang et al., 2021 [67] |
| Anhui Province | Cotton, maize, soybean, wheat | 11 | Jiao, 2013 [68] |
| Anhui Province | Cotton, maize, soybean | 6 | Jiao et al., 2009 [69] |
| Anhui Province | Cotton, maize | 3 | Jiao et al., 2012 [70] |
| Anhui Province | Rice | 2 | Jiao et al., 2007 [71] |
| Anhui Province | Rape seed, rice | 30 | Jin et al., 2021 [72] |
| Anhui Province | Rice, wheat | 18 | Li, 2023 [73] |
| Hunan Province | Maize, sweet potato | 1 | Li et al., 2011 [74] |
| Jilin Province | Maize | 6 | Li et al., 2020 [75] |
| Zhejiang Province | Rice | 4 | Li et al., 2016 [76] |
| Hunan Province | Maize, rice | 2 | Li et al., 2012 [77] |
| Guangdong Province | Vegetable | 11 | Li, 2019 [78] |
| Guangdong Province | Vegetable | 13 | Li et al., 2015 [79] |
| Hubei Province | Rice | 5 | Li, 2018 [80] |
| Anhui Province | Rice, wheat | 18 | Li et al., 2023 [81] |
| Hubei Province | Rice | 7 | Li, 2018 [82] |
| Fujian Province | Rice | 10 | Li et al., 2019 [83] |
| Hunan Province | Rice | 10 | Li, 2017 [84] |
| Yunnan Province | Other | 3 | Li et al., 2010 [85] |
| Zhejiang Province | Rice | 8 | Liang et al., 2005 [86] |
| Zhejiang Province | Rice, vegetable | 12 | Liu et al., 2019 [87] |
| Ningxia Province | Rice, wheat | 6 | Liu et al., 2012 [88] |
| Jiangsu Province | Wheat | 11 | Liu et al., 2012 [89] |
| Heilongjiang Province | Rice | 5 | Liu et al., 2018 [90] |
| Heilongjiang Province | Rice | 10 | Liu et al., 2018 [91] |
| Jiangsu Province | Other | 27 | Liu, 2020 [92] |
| Beijing | Maize | 2 | Liu et al., 2024 [93] |
| Anhui Province | Rice, wheat | 11 | Lou et al., 2020 [94] |
| Shanghai | Rice, wheat | 14 | Lu, 2007 [95] |
| Yunnan Province | Other | 31 | Lu et al., 2012 [96] |
| Hubei Province | Maize, rice | 4 | Luo and Hong, 2011 [97] |
| Henan Province | Maize, wheat | 3 | Luo et al., 2023 [98] |
| Henan Province | Vegetable | 9 | Lv et al., 2021 [99] |
| Zhejiang Province | Other | 3 | Lv et al., 2017 [100] |
| Shanghai | Rice, wheat | 14 | Mao et al., 2006 [101] |
| Jiangsu Province | Vegetable | 1 | Min and Shi, 2017 [102] |
| Jiangsu Province | Rice | 11 | Mu et al., 2020 [103] |
| Hunan Province | Vegetable | 1 | Nie et al., 2009 [104] |
| Guangdong Province | Vegetable | 2 | Ning et al., 2011 [105] |
| Anhui Province | Rice | 4 | Niu, 2021 [106] |
| Hubei Province | Rice | 12 | Peng et al., 2023 [107] |
| Shanghai | Maize, other | 2 | Qian et al., 2010 [108] |
| Jiangsu Province | Rice | 10 | Qiao et al., 2015 [109] |
| Hubei Province | Rice | 33 | Qiao, 2021 [110] |
| Hubei Province | Rice | 4 | Qiao et al., 2021 [111] |
| Guangxi Province | Peanut | 8 | Qin et al., 2016 [112] |
| Hebei Province | Maize, wheat | 9 | Qin et al., 2020 [113] |
| Jiangsu Province | Rice | 1 | Qiu et al., 2009 [114] |
| Henan Province | Rice, maize, vegetable, fruit, other | 6 | Shen et al., 2012 [115] |
| Hunan Province | Rice | 12 | Shi et al., 2010 [116] |
| Zhejiang Province | Rice | 4 | Si, 2018 [117] |
| Zhejiang Province | Other | 8 | Si, 2018 [118] |
| Yunnan Province | Other | 8 | Song et al., 2010 [119] |
| Heilongjiang Province | Maize | 13 | Su et al., 2017 [120] |
| Jiangsu Province | Rice | 3 | Sun et al., 2018 [121] |
| Shandong Province | Maize, wheat | 8 | Tan et al., 2015 [122] |
| Shandong Province | Maize, wheat | 1 | Tan et al., 2011 [123] |
| Shandong Province | Maize, wheat | 2 | Tan et al., 2011 [124] |
| Hunan Province | Rice | 2 | Tian et al., 2010 [125] |
| Shanghai | Rice | 12 | Tian, 2016 [126] |
| Henan Province | Maize, soybean | 50 | Tian et al., 2016 [127] |
| Jiangsu Province | Rice | 4 | Tian et al., 2007 [128] |
| Jiangsu Province | Rice, wheat | 37 | Tian et al., 2007 [129] |
| Hunan Province | Other | 8 | Tuo, 2015 [130] |
| Jiangsu Province | Vegetable | 9 | Wang et al., 2011 [131] |
| Jiangxi Province | Rice | 8 | Wang, 2007 [132] |
| Shanghai | Rice | 6 | Wang et al., 2018 [133] |
| Anhui Province | Rice | 1 | Wang et al., 2010 [134] |
| Anhui Province | Maize, rice | 11 | Wang et al., 2010 [135] |
| Anhui Province | Wheat | 1 | Wang et al., 2012 [136] |
| Anhui Province | Rape seed, wheat | 4 | Wang et al., 2015 [137] |
| Zhejiang Province | other | 8 | Wang et al., 2021 [138] |
| Anhui Province | Sesame, wheat | 9 | Wang et al., 2016 [139] |
| Beijing | Maize | 2 | Wang et al., 2017 [140] |
| Jiangsu Province | Vegetable, other | 4 | Wang et al., 2017 [141] |
| Hunan Province | Maize | 5 | Wang, 2015 [142] |
| Zhejiang Province | Other | 5 | Wang, 2017 [143] |
| Jilin Province | Rice | 6 | Wang, 2024 [144] |
| Jiangsu Province | Rice, wheat | 9 | Wang et al., 2007 [145] |
| Jiangsu Province | Rice | 1 | Wang et al., 2011 [146] |
| Beijing | Other | 1 | Wang et al., 2003 [147] |
| Jiangsu Province | Rice | 12 | Wang et al., 2007 [148] |
| Jiangsu Province | Rice | 6 | Wang et al., 2004 [149] |
| Jilin Province | Maize | 6 | Wang et al., 2016 [150] |
| Shandong Province | peanut | 1 | Wang et al., 2011 [151] |
| Shanghai | Rice | 2 | Wen et al., 2011 [152] |
| Zhejiang Province | Rice | 8 | Wu et al., 2012 [153] |
| Guangdong Province | Maize, vegetable | 2 | Wu and Chen, 1999 [154] |
| Jiangsu Province | Rice | 2 | Xi et al., 2009 [155] |
| Hubei Province | Rice | 9 | Xia et al., 2022 [156] |
| Hubei Province | Cotton, rape seed | 9 | Xia et al., 2014 [157] |
| Jiangsu Province | Wheat | 3 | Xiao et al., 2005 [158] |
| Guangxi Province | Other | 9 | Xie et al., 2013 [159] |
| Jiangsu Province | Rice | 1 | Xie et al., 2007 [160] |
| Beijing | Vegetable | 6 | Xu et al., 2013 [161] |
| Liaoning Province | Maize | 6 | Xu et al., 2021 [162] |
| Anhui Province | Wheat | 16 | Xu et al., 2018 [163] |
| Jiangsu Province | Rice | 6 | Xue et al., 2011 [164] |
| Jiangsu Province | Rice, wheat | 30 | Xue et al., 2014 [165] |
| Chongqing | Maize, wheat | 1 | Yan et al., 2014 [166] |
| Jiangsu Province | Other | 3 | Yan et al., 2023 [167] |
| Jilin Province | Maize | 8 | Yan, 2016 [168] |
| Jilin Province | Maize | 5 | Yan et al., 2018 [169] |
| Jilin Province | Maize | 4 | Yan et al., 2017 [170] |
| Jilin Province | Maize, wheat | 13 | Yan et al., 2014 [171] |
| Jilin Province | Maize | 10 | Yan et al., 2016 [172] |
| Shandong Province | Vegetable | 5 | Yan, 2014 [173] |
| Jiangsu Province | Rice | 4 | Yang et al., 2015 [174] |
| Shandong Province | Peanut | 5 | Yang et al., 2023 [175] |
| Hunan Province | Rice | 4 | Yang, 2011 [176] |
| Heilongjiang Province | Maize, soybean, other | 15 | Yang et al., 2018 [177] |
| Yunnan Province | Vegetable | 12 | Yang, 2022 [178] |
| Yunnan Province | Vegetable | 4 | Yang et al., 2023 [179] |
| Guangdong Province | Rice | 52 | Yao et al., 2015 [180] |
| Chongqing | Maize, wheat | 8 | Yao et al., 2010 [181] |
| Zhejiang Province | Rice | 1 | Ye et al., 2016 [182] |
| Shanghai | Rice | 6 | Yi, 2020 [183] |
| Jiangxi Province | Rice, other, vegetable | 10 | Yu et al., 2010 [184] |
| Guangxi Province | Rice | 1 | Yu, 2011 [185] |
| Zhejiang Province | Rape seed | 7 | Yu et al., 2011 [186] |
| Jiangsu Province | Wheat | 5 | Yu et al., 2011 [187] |
| Zhejiang Province | Rape seed, soybean | 3 | Yuan et al., 2002 [188] |
| Hunan Province | Rice | 7 | Yuan et al., 2021 [189] |
| Guangdong Province | Rice | 9 | Zeng et al., 2010 [190] |
| Guangdong Province | Other | 3 | Zhang, 2016 [191] |
| Chongqing | Rice | 2 | Zhang et al., 2011 [192] |
| Zhejiang Province | Rice | 3 | Zhang, 2021 [193] |
| Liaoning Province | Maize | 2 | Zhang et al., 2023 [194] |
| Zhejiang Province | Rice, vegetable | 55 | Zhang et al., 2011 [195] |
| Jiangsu Province | Rice | 6 | Zhang et al., 2018 [196] |
| Shannxi Province | Wheat, other | 10 | Zhang, 2002 [197] |
| Fujian Province | Rice, other | 26 | Zhang et al., 2012 [198] |
| Hunan Province | Maize, vegetable | 3 | Zhang et al., 2013 [199] |
| Hunan Province | Soybean | 1 | Zhang et al., 2014 [200] |
| Zhejiang Province | Rice | 8 | Zhang et al., 2001 [201] |
| Jiangsu Province | Rice | 12 | Zhao et al., 2012 [202] |
| Shaanxi Province | Rice | 2 | Zhao et al., 2006 [203] |
| Jiangsu Province | Wheat | 1 | Zhao et al., 2009 [204] |
| Jiangxi Province | Peanut | 8 | Zheng et al., 2022 [205] |
| Hubei Province | Rice | 8 | Zhou et al., 2023 [206] |
| Jiangsu Province | Rice | 2 | Zhou et al., 2019 [207] |
| Jiangsu Province | Maize, rape seed, rice, vegetable | 7 | Zhu et al., 2006 [208] |
| Hunan Province | Rice | 10 | Zhu et al., 2019 [209] |
| Jiangsu Province | Wheat, Rice | 2 | Zhu et al., 2006 [210] |

Note: Crop types mainly include rice, wheat, maize, soybean, sweet potato, cotton, peanuts, rapeseed, sesame, vegetables, and others (e.g., sugarcane, fruit, and tobacco, etc.

**Table S5. Nitrogen removal coefficient in Chinese waterbodies.**

| **Range** | **Lake or reservoir (%/ha)** | **River (%/km)** | | | |
| --- | --- | --- | --- | --- | --- |
|  |  | **First-type** | **Second-type** | **Third-type** | **Fourth-type** |
| **min** | 0.63 | 0.13 | 0.19 | 0.33 | 0.39 |
| **max** | 0.71 | 0.23 | 0.83 | 0.61 | 0.74 |

**Table S6. Nitrogen reduction efficiency by different measures.**

| **Region** | **Measure type** | **Specific measure** | **N reduction efficiency** | **Source** |
| --- | --- | --- | --- | --- |
| **Arid North** | Source-oriented | Weather-responsive fertilization | 31.6% | Zhao et al., 2020 [211] |
|  |  | Weather-responsive fertilization | 15.61% | Bian et al., 2008 [212] |
|  |  | Weather-responsive fertilization | 35.2% | Li et al., 2021 [213] |
|  |  | Weather-responsive fertilization | 52.2% | Li et al., 2021 [213] |
|  |  | Weather-responsive fertilization | 32% | Jiao et al., 2007 [214] |
|  |  | Controlled-release fertilizers | 34% | You et al., 2024 [215] |
|  |  | Controlled-release fertilizers | 20% | Xue et al., 2020 [216] |
|  |  | Controlled-release fertilizers | 25% | Xue et al., 2014 [217] |
|  |  | Controlled-release fertilizers | 51.77% | Gu et al., 2023 [218] |
|  |  | Controlled-release fertilizers | 26% | Ding et al., 2020 [219] |
|  |  | Controlled-release fertilizers | 16.86% | Hou et al., 2021 [220] |
|  |  | Controlled-release fertilizers | 48% | Li et al., 2018 [221] |
|  |  | Controlled-release fertilizers | 15% | Li et al., 2018 [221] |
|  |  | Controlled-release fertilizers | 21.25% | Su et al., 2024 [222] |
|  |  | Controlled-release fertilizers | 33.9% | Su et al., 2024 [222] |
| **Arid North** | Source-oriented | Controlled-release fertilizers | 43.7% | Su et al., 2024 [222] |
|  |  | Controlled-release fertilizers | 49.7% | Su et al., 2024 [222] |
|  |  | Controlled-release fertilizers | 31.7% | Xia et al., 2016 [223] |
|  |  | Controlled-release fertilizers | 58.29% | Shan et al., 2015 [224] |
|  |  | Controlled-release fertilizers | 61.01% | Shan et al., 2015 [224] |
|  |  | Controlled-release fertilizers | 49.33% | Shan et al., 2015 [224] |
|  |  | Controlled-release fertilizers | 56.05% | Shan et al., 2015 [224] |
|  |  | Controlled-release fertilizers | 59.79% | Shan et al., 2015 [224] |
|  |  | Controlled-release fertilizers | 63.59% | Shan et al., 2015 [224] |
|  |  | Split applications | 21% | You et al., 2024 [215] |
|  |  | Split applications | 36.5% | Xia et al., 2016 [223] |
|  |  | Split applications | 14.85% | Ren et al., 2024 [225] |
|  |  | Split applications | 16.5% | Wang et al., 2015 [226] |
|  |  | Split applications | 23.8% | Tao et al., 2024 [227] |
|  |  | Split applications | 31.25% | Han et al., 2023 [228] |
|  |  | Split applications | 20.61% | Han et al., 2023 [228] |
| **Humid South** | Process-based | Riparian wetland restoration | 7.06% | Yang and Best, 2015 [229] |
|  |  | Riparian wetland restoration | 51.37% | Fan et al., 2024 [230] |
|  |  | Riparian wetland restoration | 15.4% | Lyu et al., 2021 [231] |
|  |  | Riparian wetland restoration | 34.13% | Lyu et al., 2021 [231] |
|  |  | Riparian wetland restoration | 49.49% | Lyu et al., 2021 [231] |
|  |  | Riparian wetland restoration | 49.69% | Lyu et al., 2021 [231] |
|  |  | Riparian wetland restoration | 32.1% | Xie et al., 2023 [232] |
|  |  | Riparian wetland restoration | 9% | Zhang et al., 2024 [233] |
|  |  | Riparian wetland restoration | 38.66% | Tang et al., 2013 [234] |
|  |  | Aquatic vegetation management | 32.5% | Bai et al., 2025 [235] |
|  |  | Aquatic vegetation management | 21.4% | Shen et al., 2021 [236] |
|  |  | Aquatic vegetation management | 17% | Yan et al., 2024 [237] |
|  |  | Aquatic vegetation management | 12.4% | Zhao et al., 2012 [238] |
|  |  | Aquatic vegetation management | 19.7% | Cui et al., 2022 [239] |
|  |  | Aquatic vegetation management | 36.6% | Zhang et al., 2024 [240] |
|  |  | Aquatic vegetation management | 14.8% | Shen et al., 2024 [241] |
| **Humid South** | Process-based | Aquatic vegetation management | 16.49% | Ge et al., 2016 [242] |
|  |  | Aquatic vegetation management | 23.46% | Ge et al., 2016 [242] |
|  |  | Aquatic vegetation management | 7.2% | Wu et al., 2017 [243] |
|  |  | Aquatic vegetation management | 12% | Zhang et al., 2022 [244] |
|  |  | Aquatic vegetation management | 26.62% | Yin et al., 2025 [245] |
|  |  | Aquatic vegetation management | 28.05% | Yin et al., 2025 [245] |
|  |  | Aquatic vegetation management | 10.35% | Du et al., 2018 [246] |
|  |  | Aquatic vegetation management | 50.9% | Zhang et al., 2016 [247] |
|  |  | Aquatic vegetation management | 36.3% | Zhang et al., 2016 [247] |
|  |  | Aquatic vegetation management | 12.2% | Fan et al., 2024 [240] |
|  |  | Aquatic vegetation management | 12.43% | Fan et al., 2024 [240] |
|  |  | Aquatic vegetation management | 19.5% | Wang et al., 2020 [248] |
|  |  | Aquatic vegetation management | 12.4% | Wang et al., 2020 [248] |
|  |  | Decentralized retention ponds | 21% | Shen et al., 2025 [249] |
|  |  | Decentralized retention ponds | 12% | Zhang et al., 2019 [250] |
|  |  | Decentralized retention ponds | 12.7% | Wang et al., 2025 [251] |
| **Humid South** | Process-based | Decentralized retention ponds | 26.39% | Wang et al., 2025 [251] |
|  |  | Decentralized retention ponds | 7.2% | Lyu et al., 2024 [252] |
|  |  | Decentralized retention ponds | 39% | Li et al., 2020 [253] |
|  |  | Decentralized retention ponds | 23.3% | Li et al., 2019 [254] |
|  |  | Decentralized retention ponds | 58.71% | Tang et al., 2013 [234] |
|  |  | Decentralized retention ponds | 40.74% | Wang et al., 2022 [255] |
|  |  | Decentralized retention ponds | 44.3% | Li et al., 2020 [256] |
|  |  | Decentralized retention ponds | 11.56% | Beusen et al., 2016 [257] |
|  |  | Decentralized retention ponds | 4.18% | Li et al., 2023 [258] |
|  |  | Decentralized retention ponds | 22.74% | Sun et al., 2021 [259] |

**Fig. S1. Global distribution of climate zones and projected precipitation in 2050.**

**a**, Global distribution of humid, arid and semi-arid regions based on the aridity index. **b**, Projected global precipitation for 2050.

**Fig. S2. Spatial patterns of nitrogen runoff losses drivers in China.**

Distribution of soil physicochemical properties (**a**–**c**), including soil total nitrogen content (**a**), pH (**b**), and clay content (**c**). Distribution of mean annual precipitation (**d**) and temperature (**e**). Distribution of nitrogen fertilizer application in paddy fields (**f**) and drylands (**g**).

**Fig. S3. Spatial distribution of nitrogen runoff losses from croplands in 2019.**

**a**–**b**, Distribution of total N runoff background (**a**) and fertilizer runoff losses (**b**) from paddy fields. **c**, Percentage of N runoff from paddy fields and drylands in arid northern and humid southern regions. **d**–**e**, Distribution of total N runoff background (**d**) and fertilizer runoff losses (**e**) from drylands.

**Fig. S4. Threshold response of N runoff intensity to annual precipitation in the arid North.**

Segmented regression was performed between annual precipitation and N runoff intensity using 10,000 randomly selected paired raster samples from the arid North in each repetition. The dashed line indicates the mean precipitation threshold from 50 repeated random samples, and the shaded band indicates the corresponding 95% range. Across repeated samples, the mean *R*^2^ was 0.20, and the slope-change term was significant in all cases (*P* < 0.001). The relatively modest *R*^2^ reflects unresolved sub-grid heterogeneity in soil properties, land use, and management practices, and confirms that the precipitation threshold represents the dominant rather than exclusive control on N runoff at the grid scale. The identified threshold (~457 mm) falls within the semi-arid to sub-humid transition zone of northern China, where ephemeral drainage networks become hydrologically activated.

**Fig. S5. Relationship between annual total precipitation and extreme rainfall days in arid northern China (2015**–**2024).**

Extreme days are defined as daily precipitation > 50 mm.

**Fig. S6. Removal of cropland nitrogen runoff along the waterways in 2019.**

Removal of cropland nitrogen runoff from lakes /reservoirs (**a**), and rivers (**b**).

**Fig. S7. Nitrogen transport and water network spatial patterns.**

**a**, Average nitrogen travel length and removal percentage. **b**, Water network density.

**Fig. S8. Annual precipitation and temperature for two scenarios in 2050.**

Annual precipitation under SSP2-4.5 (**a**) and SSP5-8.5 scenarios (**b**). Annual temperature under SSP2-4.5 (**c**) and SSP5-8.5 scenarios (**d**).

**Fig. S9. Changes in cropland nitrogen runoff to the ocean by 2050 under SSP2-4.5 and SSP5-8.5.**

Predicted impacts of precipitation and temperature on absolute changes in cropland nitrogen runoff to the ocean by 2050 under SSP2-4.5 (**a**) and SSP5-8.5 (**b**).

**Fig. S10.** **Nitrogen removal and transport through water systems across 10 Chinese river basins under two climate scenarios in 2050.**

Nitrogen removal and transport through water systems across 10 Chinese river basins by 2050 under SSP2-4.5 (**a**) and SSP5-8.5 (**b**). HA, Hai River Basin; HU, Huai River Basin; YL, Yellow River Basin; SH, Songhua River Basin; LH, Liao River Basin; NW, Northwest River Basin; PL, Pearl River Basin; SW, Southwest River Basin; YT, Yangtze River Basin; SE, Southeast River Basin.

**Fig. S11. Spatial delineation of the 10 Chinese river basins and distribution of study sites included in the nitrogen runoff loss database.**

**Fig. S12. An a-priori path model of background emission and fertilizer emissions of nitrogen runoff driving factors.**

The model includes soil total nitrogen content (TN), nitrogen fertilizer input (N_fert_), mean annual precipitation (MAP), soil clay content (Clay), soil pH value (pH), mean annual temperature (MAT), total nitrogen background runoff (NBL*_runoff_*), total nitrogen fertilizer runoff (NFL*_runoff_*).

**Fig. S13. Comparison of observed and simulated nitrogen runoff losses in paddy fields and drylands in China. Solid lines represent 1:1 (y = x) line.**

Validation of data incorporated into the model (a). Remaining data validation (b). PB, background emissions from paddy field; PF, fertilizer emissions from paddy field; DB, background emissions from dryland; DF, fertilizer emissions from dryland.

**Fig. S14. Cumulative travel paths in water bodies for each grid source in China.**

Cumulative travel area in lakes/reservoirs (**a**). Cumulative travel length in first-type river (**b**), second-type river (**c**), third-type river (**d**), and fourth-type river (**e**).

**Fig. S15. Distribution of freshwater systems in China.**

Lake/reservoir (**a**). The 1st–3rd, 4th and 5th order river (**b**).

**References**

1. Zhao D, Qiu J, Fan Z *et al.* Global warming has imbalance impact on soil nitrogen transformation rates. *Earth's Future* 2025; **13**: e2024EF004756.
2. Xia Y, Zhao D, Yan X *et al.* A new framework to model the distributed transfer and retention of nutrients by incorporating topology structure of small water bodies. *Water Research* 2023; **238**: 119991.
3. Deng O, Huang S, Wang C *et al.* Atmospheric nitrogen pollution control benefits the coastal environment. *Environmental Science & Technology* 2023; **58**: 449-58.
4. Zhu Z, Zhang X, Dong H *et al.* Integrated livestock sector nitrogen pollution abatement measures could generate net benefits for human and ecosystem health in China. *Nature Food* 2022; **3**: 161-8.
5. Qiu J, Zhao D, Hu W *et al.* Revealing the overestimation of phosphorus fertilizer runoff to aquatic phosphorus pollution in China. *Resources, Conservation and Recycling* 2026; **225**: 108626.
6. Gao Y, Zhou F, Ciais P *et al.* Human activities aggravate nitrogen-deposition pollution to inland water over China. *National Science Review* 2020; **7**: 430-440.
7. He J, Hao C. Analysis on spatial-temporal variation characteristics of climate in Qinling-Huaihe demarcation zone since 1961. *Ecological Indicators* 2024; **158**: 111345.
8. Michalak AM. Study role of climate change in extreme threats to water quality. *Nature* 2016; **535**: 349-50.
9. Ockenden MC, Hollaway MJ, Beven KJ *et al.* Major agricultural changes required to mitigate phosphorus losses under climate change. *Nature Communications* 2017; **8**: 161.
10. Shen W, Li S, Basu NB *et al.* Size and temperature drive nutrient retention potential across water bodies in China. *Water Research* 2023; **239**: 120054.
11. Keller AA, Chen X, Fox J *et al.* Attenuation coefficients for water quality trading. *Environmental Science & Technology*. 2014; **48**: 6788-94.
12. Chapra SC. Surface water-quality modeling. *Waveland press* 2008.
13. Cheng FY, Basu NB. Biogeochemical hotspots: Role of small water bodies in landscape nutrient processing. *Water Resources Research* 2017; **53**: 5038-56.
14. Ferro V, Porto P. Sediment delivery distributed (SEDD) model. *Journal of hydrologic engineering* 2000; **5**: 411-22.
15. You L, Ros GH, Chen Y *et al.* Optimized agricultural management reduces global cropland nitrogen losses to air and water. *Nature Food* 2024; **5**: 995-1004.
16. Tang W, Zhang W, Zhao Y *et al.* Nitrogen removal from polluted river water in a novel ditch–wetland–pond system. *Ecological Engineering* 2013; **60**: 135-9.
17. Li D, Chu Z, Li P *et al.* Impacts of landscape spatial configuration of integrated multi-pond constructed wetlands in a basin on the treatment of non-point source pollution. *Journal of Cleaner Production* 2023; **383**: 135389.
18. Mulholland PJ, Helton AM, Poole GC et al. Stream denitrification across biomes and its response to anthropogenic nitrate loading. *Nature* 2008; **452**: 202–5.
19. Bodirsky BL, Popp A, Lotze-Campen H et al. Reactive nitrogen requirements to feed the world in 2050 and potential to mitigate nitrogen pollution. *Nature Communications* 2014; **5**: 3858.
20. Mogollón JM, Lassaletta L, Beusen AHW et al. Assessing future reactive nitrogen inputs into global croplands based on the shared socioeconomic pathways. *Environmental Research Letters* 2018; **13**: 044008.
21. Bu Q, Fan X, Tian Y *et al.* Effect of different application level of N on the forms and losing amount of N in the runoff of cotton field. *Hubei Agricultural Sciences* 2009; **48**: 1092-5.
22. Cao Y, Fu Z, Sun H *et al.* Effects of N application rates on N use efficiency by rice and N runoff loss. *Soils* 2016; **48**: 868-72.
23. Chen J, Huang C, Zheng M *et al.* Effects of substituting organic fertilizer and biochar-based fertilizer instead of chemical fertilizer on nitrogen and phosphorus runoff loss in sweet potato sloping farmland. *Journal of Zhejiang A&F University* 2023; **40**: 540-9.
24. Chen J, Jiang N, Liu X *et al.* Effects of controlled-release nitrogen fertilizer reduction on crop yield, nitrogen absorption and runoff loss. *Acta Agriculturae Zhejiangensis* 2024; **36**: 846-58.
25. Chen Q, Xi Y, Wang L *et al.* Characteristics of nitrogen and phosphorus runoff losses in organic and conventional rice-wheat rotation farmland in Taihu Lake Region. *Journal of Agro-Environment Science* 2016; **35**: 1550-8.
26. Chen S, Meng F, Wu W *et al.* Nitrogen loss characteristics via runoff in typical rice planting area in Northeast China under different planting managements. *Chinese Journal of Eco-Agriculture* 2012; **20**: 728-33.
27. Chen Y, Zhao L, Yang Y *et al.* Preliminary study on characteristics of nitrogen and phosphorus runoff losses from rice fields in Hai River Basin, China. *Journal of Agro-Environment Science* 2011; **30**: 328-33.
28. Chen Z, Ying S, Liu Y *et al.* Effects of different fertilizer types on nitrogen loss in paddy field. *Journal of Soil and Water Conservation* 2021; **35**: 36-43.
29. Chen Z, Zhu B, Liu X. Characteristics of nitrogen transfer with runoff on sloping cropland of purple soil under natural rainfall. *Yangtze River* 2014; **45**: 82-85.
30. Chu L, Wang K, Bai W *et al.* Impact of level terrace on runoff, sediment and N&P loss from sloping filed. *Journal of Research Soil and Water Conservation* 2010; **24**: 1-6.
31. Cong Z, Li N. The impact of agricultural tillage practices on the interception of surface runoff pollutants on sloping farmland. *Technology of Soil and Water Conservation* 2021: 22-4.
32. Deng Q, Fan M, Zhu J. Characteristics of nitrogen loss from drainage and runoff in paddy field and rainfall runoff. *Hubei Agricultural Sciences* 2022; **61**: 17-22.
33. Dong X. Effect of crop kinds on runoff and nitrogen losses of slope farmland. *Journal of Soil and Water Conservation* 2016; **30**: 56-61.
34. Duan L, Duan Z, Chang J. Effect of surface management and fertilization mode on nitrogen runoff from upland in Taihu Lake Region. *Journal of Agro-Environment Science* 2007: 813-8.
35. Duan R. Effect of different fertilizer application methods on nitrogen transformations in paddy soils and microbial mechanisms. Chinese Academy of Agricultural Sciences Dissertation, Chinese Academy of Agricultural Sciences Dissertation, 2018.
36. Duan R, Tang Y, Wang Y *et al.* Effects of different fertilization modes on rice yield and nitrogen loss in paddy soils under double cropping rice. *Chinese Journal of Eco-Agriculture* 2017; **25**: 1815-22.
37. Duan R, Tang Y, Wen J *et al.* Effect of reducing fertilizer application on crop yield and nitrogen and phosphorus loss in runoff from embankment upland in Dongting Lake Region. *Chinese Journal of Eco-Agriculture* 2013; **21**: 536-43.
38. Fan C, Luo Y, Wang W *et al.* Effects of different management practices on nitrogen runoff losses from sloping yellow soil. *Journal of Agro-Environment Science* 2014; **33**: 1948-55.
39. Fan H. Comparison of active nitrogen (N2O, NH3, NO3--N) losses among different new nitrogen fertilizers treatments in cropland. Nanjing Agricultural University, Nanjing Agricultural University, 2016.
40. Fan H, Xu L, Zhao X *et al.* Study on Nitrogen Loss in Rice-wheat Rotation Farmland in Taihu Basin. *Ecology and Environmental Sciences* 2015; **24**: 255-62.
41. Fu B, Meng Q, Qiu Y *et al.* Effects of land use on soil erosion and nitrogen loss in the hilly area of the Loess Plateau, China. *Land Degradation & Development* 2004; **15**: 87-96.
42. Gao D, Liu Q, Zhang Y *et al.* Effects of adding biochar on yield, quality, fertilizer utilization of summer maize and nitrogen and phosphorus runoff losses from field. *Soil and Fertilizer Sciences in China* 2015: 72-6.
43. Gao D, Zhang L, Liu Q *et al.* Effects of different fertilization modes on soil nitrogen runoff in dryland field. *Journal of Soil and Water Conservation* 2014; **28**: 209-13.
44. Gao J, He R, Yu L *et al.* Research on water quality and runoff loss of nitrogen and phosphorus from the typical agricultural watersheds in three gorges reservoir area——A case study in Huolong, Yunyang County. *Environment and Ecology in the Three Gorges* 2012; **34**: 6-12.
45. Gong J, Jin Y, Hu H *et al.* Effects of rice straw incorporation on nitrogen, phosphorus and COD loss in rape seasonal runoff. *Journal of Soil and Water Conservation* 2019; **33**: 24-9.
46. Gu J, Li Y, Yang L *et al.* Research on the dynamic change and runoff loss of nitrogen in the soil surface water of rice fields of direct seeding cultivation. *Journal of Anhui Agricultural Sciences* 2009; **37**: 3626-8.
47. Gu X, Wang Y, Sun Y *et al.* Increasing transplanting density and reducing nitrogen fertilizer: effect on nitrogen loss and nitrogen use efficiency in paddy field. *Chinese Agricultural Science Bulletin* 2021; **37**: 64-70.
48. Gu X, Wang Y, Zhang L *et al.* Effects of Fertilization and Transplanting Integrated Technology on Rice Yield and Nitrogen Loss. *Heilongjiang Agricultural Sciences* 2017: 12-7.
49. Guo L, Luo M, Chang J *et al.* Effects of organic nutrient replacement on wheat yield, soil fertility and runoff loss of nitrogen and phosphorus in wheat field. *Journal of Nuclear Agricultural Sciences* 2022; **36**: 2063-71.
50. Guo Y, Wang C, Wang H *et al.* Effects of fertilization management on flue-cured tobacco yield，quality and loss of nitrogen and phosphorus runoff in Erhai Lake Basin. *Soil and Fertilizer Sciences in China* 2023: 101-9.
51. Guo Z, Liu H, Zhang Y *et al.* Effects of different fertilization modes on nitrogen loss by surface runoff and the apparent nitrogen balance in the vegetable fields of Taihu Lake Region, China. *Journal of Soil and Water Conservation* 2018; **32**: 37-42+50.
52. Guo Z, Xiao M, Chen L *et al.* Effect of the sediment by surface runoff on nitrogen loss in the paddy field. *Chinese Journal of Soil Science* 2011; **42**: 1219-24.
53. Guo Z, Zhou W, Chen L *et al.* Effects of nitrogen fertilizer application and no tillage on nitrogen loss by surface runoff during the wheat growing season in intensive rice-wheat rotation field. *Ecology and Environmental Sciences* 2011; **20**: 1253-8.
54. He B, Guo T, Yao J *et al.* Characteristics of N and P losses in sediment of purple soil on sloping farmland with different fertilization levels. *Journal of Southwest University (Natural Science Edition)* 2012; **34**: 1-8.
55. He T, Qin F, Su L *et al.* Fertilization and run-off loss of nutrient of major economic crops in sloping farmland of Guangxi Province, China. *Journal of Agriculture Resources and Environment* 2014; **31**: 169-74.
56. He X. Effects of agricultural measures on crop yield, nutrient absorption and loss. Hunan Agricultural University, Hunan Agricultural University, 2017.
57. Hou X, Gong J, Miao J *et al.* Economic and environmental benefits of straw returning to field and chemical fertilizer combination. *Journal of Honghe University* 2019; **17**: 130-3.
58. Huang D, Li W, Wang L *et al.* Effects of water and fertilizer managements on yield, nutrition uptake of rice and of nitrogen and phosphorus loss of runoff from paddy field. *Journal of Soil and Water Conservation* 2013; **27**: 62-6.
59. Huang S. Effects of different fertilization modes on nitrogen and phosphorus loss in double-cropping rice. Hunan Agricultural University, Hunan Agricultural University, 2020.
60. Huang S, Lu Y, Shen G *et al.* Nitrogen losses from dry farming fields in Shanghai suburbs. *Rural Eco-environment* 2005: 50-3.
61. Huang T, Rong X, Liu Q *et al.* Effects of different organic fertilization modes on yield, quality, fertilizer nitrogen utilization of spring maize and nitrogen loss from field. *Soils* 2010; **42**: 915-9.
62. Huang Z, Zheng X, Yao C. The study of nonirrigated farmland nitrogen and phosphorus loss with surface runoff in Shanghai. *Yunnan Geographic Environment Research* 2007: 6-10.
63. Huo H, Wang T, Wei S *et al.* Characteristics of nitrogen loss from hillslope croplands of purple soil in the Three Gorges Reservoir area and impacts of slope gradients. *Journal of Southwest University (Natural Science Edition)* 2013; **35**: 112-7.
64. Ji X, Zheng S, Lu Y *et al.* Dynamics of floodwater nitrogen and its runoff loss, urea and controlled release nitrogen fertilizer application regulation in rice. *Scientia Agricultura Sinica* 2006: 2521-30.
65. Ji X, Zheng S, Lu Y *et al.* Effects of controlled release nitrogen fertilizer on surface water N dynamics and its runoff loss in double cropping paddy fields in Dongtinghu Lake are. *Chinese Journal of Applied Ecology* 2007: 1432-40.
66. Ji X, Zheng S, Lu Y *et al.* Study of dynamics of floodwater nitrogen and regulation of its runoff loss in paddy field-based two-cropping rice with urea and controlled release nitrogen fertilizer application. *Agricultural Sciences in China* 2007: 189-99.
67. Jiang H, Shen S, Gu Y *et al.* Effects of different fertilization treatments on runoff losses of nitrogen and phosphorus in paddy fields in Erhai Lake basin, China. *Journal of Agro-Environment Science* 2021; **40**: 1305-13.
68. Jiao P. Evaluation of nitrogen and phosphorus losses by surface runoff with experiment and simulation in Huaibei Plain. China Institute of Water Resources and Hydropower Research, China Institute of Water Resources and Hydropower Research, 2013.
69. Jiao P, Wang S, Xu D *et al.* Effect of crop vegetation type on nitrogen and phosphorus runoff losses from farmland in one rainstorm event. *Journal of Hydraulic Engineering* 2009; **40**: 296-302.
70. Jiao P, Xu D, Wang S *et al.* Nitrogen loss by surface runoff from summer crops in Huainbei Plains affected by fertilization modes. *Ecology and Environmental Sciences* 2012; **26**: 64-8.
71. Jiao S, Hu X, Pan G *et al.* Effects of fertilization on nitrogen and phosphorus run-off loss from Qingzini paddy soil in Taihu Lake region during rice growth season. *Chinese Journal of Ecology* 2007: 495-500.
72. Jin Y, Liu Y, Hu H *et al.* Effects of continuous straw returning with chemical fertilizer on annual runoff loss of nitrogen and phosphorus in rice-rape rotation. *Scientia Agricultura Sinica* 2021; **54**: 1937-51.
73. Li C. Effects of organic nutrient iso-nitrogen substitution on rice and wheat yield and nitrogen and phosphorus loss in Chaohu Lake. Anhui Agricultural University, Anhui Agricultural University, 2023.
74. Li E, Qiu Y, Peng P *et al.* The ecological interception technology and effects on maize yield and surface runoff in red soil slopes of Dongting Lake. *Chinese Agricultural Science Bulletin* 2011; **27**: 137-42.
75. Li F, Han X, Ma X *et al.* Straw mulch controls runoff and nitrogen and phosphorus loss from slope farmland in black soil region of Northeast China. *Journal of Soil and Water Conservation* 2020; **34**: 37-42.
76. Li J, Li S, Wu Q *et al.* Effects of different fertilization treatments on runoff and leaching losses of nitrogen in paddy field. *Journal of Soil and Water Conservation* 2016; **30**: 23-28+33.
77. Li K, Sima X, Ding S *et al.* Effects of controlled release fertilizer on loss of nitrogen and phosphorus from farmland. *Journal of Anhui Agricultural Sciences* 2012; **40**: 12466-70.
78. Li M. Study on loss characteristics of nitrogen and phosphorus in surface runoff from perennial vegetable field under different nutrition management measures. South China Agricultural University, South China Agricultural University, 2019.
79. Li M, Yao J, Wang R *et al.* Effects of different nutrition management on vegetable growth and runoff characteristics of soil nitrogen in perennial vegetable field. *Journal of Plant Nutrition and Fertilizers* 2015; **21**: 1190-1199.
80. Li P. Effect of controlled-release urea on yield, nitrogen losses and nitrogen use efficiency for double-cropping rice system. Huazhong Agricultural University, Huazhong Agricultural University, 2018.
81. Li P, Yuan T, Dong J *et al.* Analysis of the impact of conservation tillage on nitrogen loss in surface runoff and soil erosion. *Technical Supervision in Water Resources* 2023: 132-134+159.
82. Li W. Effects of different double-cropping-rice-based rotations on rice yield and nutrient runoff loss in paddy field. Hunan Agricultural University, Hunan Agricultural University, 2018.
83. Li W, Luo X, Huang M *et al.* The feature of nitrogen loss in typical rice fields in hilly areas of Fujian Province——taking Wuyishan and Minqing as examples. *Anhui Agricultural Science Bulletin* 2019; **25**: 99-102.
84. Li X. Effects of reduction application of controlled-release nitrogen fertilizer on yield, nitrogen use efficiency and nitrogen loss in paddy soil. Hunan Agricultural University, Hunan Agricultural University, 2017.
85. Li Y, Wang K, Song W *et al.* Nitrogen loss in the farmland surface runoff under the natural rainfall conditions. *Research of Soil and Water Conservation* 2010; **17**: 19-23.
86. Liang X, Tian G, Li H *et al.* Study on characteristic of nitrogen and phosphorus loss from rice field by natural rainfall runoff. *Journal of Soil and Water Conservation* 2005: 59-63.
87. Liu C, Zhang L, Lin Y *et al.* Characteristics of nitrogen and phosphorus loss by runoff from paddy and vegetable fields under different fertilization modes in Tiaoxi catchment. *Acta Agriculturae Zhejiangensis* 2019; **31**: 297-306.
88. Liu H, Zheng J, Chen L *et al.* Effects of straw-returning on annual overland runoff NPK loss in farmland. *Ecology and Environmental Sciences* 2012; **21**: 1031-6.
89. Liu R, Li Y, Zhang A *et al.* Effect of postponing n application on rice yield and n losses in Yellow River irrigation area. *Journal of Soil and Water Conservation* 2012; **26**: 16-20.
90. Liu R, Wang F, Wang K *et al.* Effects of fertilizers on nitrogen and phosphorus leaching and yield of rice in Northeast China. *Journal of Irrigation and Drainage* 2018; **37**: 63-8.
91. Liu R, Wang F, Wang K *et al.* Effects of side strip application of controlled release nitrogen fertilizer on rice yield and nitrogen loss in Northeast China. *Journal of Soil and Water Conservation* 2018; **32**: 252-6.
92. Liu T. Status of nitrogen and phosphorus non-point source pollution in Tianmu Lake. Journal of Zhejiang Agricultural Sciences, Journal of Zhejiang Agricultural Sciences, 2020.
93. Liu Z, Li H, Tao Z *et al.* Effects of natural rainfall and application of slow-release fertilizers on nitrogen and phosphorus runoff loss in the upper Beiyun River watershed. *Chinese Agricultural Science Bulletin* 2024; **40**: 74-9.
94. Lou Y, Zhu G, Zhao H *et al.* Study on characteristics of nitrogen and phosphorus loss in runoff of wheat-rice rotation in Anhui Eastern Area. *Journal of Anhui Agricultural Sciences* 2020; **48**: 78-80.
95. Lu M. Nitrogen recycling in rice-wheat rotation farm system and water environment effect. East China Normal University, East China Normal University, 2007.
96. Lu Y, Hu W, Lei B *et al.* The monitoring of nitrogen and phosphorus loss by surface runoff in sloping red soil fields of Yunnan Province, China. *Journal of Agro-Environment Science* 2012; **31**: 1544-53.
97. Luo W, Hong L. Characteristics of Nitrogen Losses in Surface Runoff from Farmland. *Journal of Irrigation and Drainage* 2011; **30**: 53-6.
98. Luo X, Kou C, Guo H *et al.* Effects of chemical fertilizer reduction and water saving on nitrogen and phosphorus runoff loss in winter wheat and summer maize rotation in aquic soil. *Soil and Fertilizer Sciences in China* 2023: 14-9.
99. Lv H, Luo X, Kou C *et al.* Effect of chemical fertilizer substitution by organic fertilizer and water saving irrigation on runoff loss of nitrogen and phosphorus in vegetable field. *Journal of Henan Agricultural Sciences* 2021; **50**: 74-80.
100. Lv T, Liao M, Ye Z *et al.* Characteristics of nitrogen runoff loss under different land uses in a rain collection area of the Hexi Reservoir in Changxing County. *Journal of Agro-Environment Science* 2017; **36**: 1369-77.
101. Mao G, Lu M, Huang M *et al.* Nitrogen loss in rice-wheat cropping farmland and its control measures. *Acta Agriculturae Shanghai* 2006: 86-92.
102. Min J, Shi W. Nitrogen discharge pathways in vegetable production as non-point sources of pollution and measures to control it. *Science of The Total Environment* 2017; **613-614**: 123.
103. Mu C, Li Z, Ouyang X *et al.* Research on the patterns and mechanisms of nitrogen and phosphorus loss in farmlands in the Taihu Lake Region. *Journal of Green Science and Technology* 2020: 134-40.
104. Nie J, Liao Y, Xie J *et al.* Impacts of biological hedgerow of vetiver on surface runoff and nitrogen loss in vegetable field under natural rainfall. *Journal of Soil and Water Conservation* 2009; **23**: 12-16+67.
105. Ning J, Xu P, Yang S *et al.* Effects of combined application of organic and inorganic fertilizer on soil nitrogen runoff in vegetable field. *Journal of Soil and Water Conservation* 2011; **25**: 17-21.
106. Niu J. Reducing nitrogen runoff loss from paddy fields by application of astragalus sinicus L. and chemical fertilizer. *Anhui Agricultural Science Bulletin* 2021; **27**: 96-99+107.
107. Peng R, Zhu J, Wu Q *et al.* Nitrogen loss and utilization characteristics of different N fertilizer translocations in direct-seeded rice. *Journal of Agriculture Resources and Environment* 2023; **40**: 651-9.
108. Qian X, Shen G, Huang L *et al.* Characteristics of nitrogen and phosphorus losses with rainfall–runoff from sandy dry field in Chongming Dongtan. *Journal of Soil and Water Conservation* 2010; **24**: 11-4.
109. Qiao J, Tang F, Zhu L *et al.* Responses of rice yield, rhizospheric soil mineral N and N runoff loss to fertilizer N in the Tai Lake Region. *Journal of Agro-Environment Science* 2015; **34**: 2140-5.
110. Qiao Y. Sources of nitrogen fertilizer and nitrogen ratio of base and topdressing on nitrogen use of direct-seeding rice. Yangtze University, Yangtze University, 2021.
111. Qiao Y, Zhu J, Wu Q *et al.* Nitrogen loss from surface runoff and ammonia volatilization from paddy field as impacted by different fertilizers and planting methods. *Journal of Irrigation and Drainage* 2021; **40**: 32-41.
112. Qin F, He T, Su L *et al.* Study on runoff and nutrient losses of soils on sloping land in wet season in southern China. *Research of Soil and Water Conservation* 2016; **23**: 19-22+27. d
113. Qin X, Pan J, Guo S *et al.* Effects of chemical fertilizer reduction combined with biogas fertilizer on crop yield of wheat–maize rotation and soil nitrogen loss in North China Plain. *Journal of Agro-Environment Science* 2020; **39**: 1558-67.
114. Qiu D, Shen S, Liu M *et al.* Study on the forms of nitrogen and phosphorus loss during the growth period of rice. *Shanghai Agricultural Science and Technology* 2009: 21-3.
115. Shen L, Miao L, Han M *et al.* Characteristics analysis of nitrogen and phosphorus loss about different land use types of Huaihe River Basin in Henan Province. *Journal of Soil and Water Conservation* 2012; **26**: 77-80.
116. Shi L, Ji X, Li H *et al.* Nitrogen and phosphorus losses from surface runoff under different application in the double cropping rice fields in Hunan. *Chinese Journal of Agrometeorology* 2010; **31**: 551-7.
117. Si L. Effects of biochar combined with fertilizer on nutrient use and loss in paddy fields. Zhejiang University, Zhejiang University, 2018.
118. Si Y. Studies on the techniques for controlling nitrogen/phosphorus losses from tea garden or bamboo forest and reduced chemical nitrogen fertilization in rice-rice cropping system. Zhejiang University, Zhejiang University, 2018.
119. Song Y, Wang K, Dong L *et al.* Dynamic changes feature of nitrogen exports via overland runoff in sloping filed in the middle regions Yunnan Province. *Journal of Soil and Water Conservation* 2010; **24**: 7-11.
120. Su M, Wang X, Gao H. Effects of tillage measures and nitrogen fertilizers on surface runoff, soil erosion and inorganic nitrogen loss in sloping land of black soil region. *Journal of Soil and Water Conservation* 2017; **31**: 58-65+163.
121. Sun G, Zhang L, Zhou W *et al.* Characteristics of nitrogen,phosphorus and potassium runoff loss in high-yielding paddy fields with continuous application of pig manure organic manure. *Jiangsu Agricultural Sciences* 2018; **46**: 349-51.
122. Tan D, Jiang L, Tan S *et al.* Effects of fertilization controlling nitrogen and phosphorus loss from farmland under wheat-maize rotation in Nansi Lake Region. *ACTA PEDOLOGICA SINICA* 2015; **52**: 128-37.
123. Tan D, Jiang L, Zhang Q *et al.* In situ study on influences of different fertilization patterns on inorganic nitrogen losses through leaching and runoff:a case of field in Nansi Lake Basin. *Acta Ecologica Sinica* 2011; **31**: 3488-96.
124. Tan D, Jiang L, Zhang Q *et al.* Characteristics of nutrient runoff losses in farmland of Nansi Lake basin under different fertilization patterns. *Journal of Plant Nutrition and Fertilizers* 2011; **17**: 464-71.
125. Tian F, Ji X, Shi L *et al.* Effects of nitrogen-reducing of different slow/controlled release fertilizer on nitrogen uptake by rice and runoff loss from double rice field in Dongting Lake area. *Research of Agricultural Modernization* 2010; **31**: 220-3.
126. Tian S. The study on loss of nitrogen and phosphorous from paddy field and construction of ecological ditch. Shanghai Jiao Tong University, Shanghai Jiao Tong University, 2016.
127. Tian Y, Li D, Wang N *et al.* Changes of runoff, sediment, nitrogen and phosphorus exported from Taowan Watershed, Funiu Mountainous Area. *Journal of Northwest Forestry University* 2016; **31**: 142-7.
128. Tian Y, Yin B, He F *et al.* Nitrogen loss with runoff in rice season in the Taihu Lake region, China. *ACTA PEDOLOGICA SINICA* 2007: 1070-5.
129. Tian Y, Yin B, Yang L *et al.* Nitrogen runoff and leaching losses during rice-wheat rotations in Taihu Lake Region, China. *Pedosphere* 2007; **17**: p.445-56.
130. Tuo H. Combining application of organic and inorganic fertilizer and straw mulching on nitrogen and phosphorus runoff losses in south-central hilly tea garden. Hunan Agricultural University, Hunan Agricultural University, 2015.
131. Wang C, Jiang Z, Zhao Y. Nitrogen and phosphorus runoff in the typical vegetable fields in Tai Lake Basin. *Journal of Soil and Water Conservation* 2011; **25**: 36-40.
132. Wang G. Study on the paddy nitrogen balance under different N and water in typical red soil areas. Nanjing Agricultural University, Nanjing Agricultural University, 2007.
133. Wang J, Fu Z, Chen G *et al.* Runoff nitrogen (N) losses and related metabolism enzyme activities in paddy field under different nitrogen fertilizer levels. *Environmental Science and Pollution Research International* 2018.
134. Wang J, Guo X, Wang Y. Effect of straw mulch on nitrogen and phosphorus loss from farmlands in Chaohu Lake Region under natural rainfall condition. *Chinese Journal of Eco-Agriculture* 2010; **18**: 492-5.
135. Wang J, Guo X, Wang Y *et al.* Effects of conservation tillage and balanced fertilizer application on nitrogen loss from paddy field and rice yields in Chaohu region of Anhui Province. *Journal of Agro-Environment Science* 2010; **29**: 1164-71.
136. Wang J, Guo X, Wang Y *et al.* Characteristics of nutrient runoff losses from farmland under different tillage and fertilization methods in Chaohu Lake region. *Journal of Soil and Water Conservation* 2012; **26**: 6-11.
137. Wang J, Lv G, Guo X *et al.* Conservation tillage and optimized fertilization reduce winter runoff losses of nitrogen and phosphorus from farmland in the Chaohu Lake region, China. *Nutrient Cycling in Agroecosystems* 2015; **101**: 93-106.
138. Wang J, Wang X, Si Y *et al.* Effects of balanced reduced fertilization and inter-row planting on nitrogen and phosphorus loss in white tea garden. *Journal of Soil and Water Conservation* 2021; **35**: 69-76.
139. Wang J, Wang Y, Guo X *et al.* Effects of different agronomic measures on runoff, sediment and nitrogen losses from sloping cropland around Chaohu Lake. *Journal of Soil and Water Conservation* 2016; **30**: 38-43+48.
140. Wang L, Yang Y, Zheng J *et al.* Effects of fertilization on nitrogen and phosphorous losses from cropland in Beijing Mountainous Area:A case study on the Miyun Reservoir Watershed. *science of soil and water conservation* 2017; **15**: 97-104.
141. Wang Q, Wu Y, Luo L. N-loss-control-oriented readjustment of planting structure and its matching ecological compensation measures—a case study of Zhushanwan Catchment. *Acta Pedologica Sinica* 2017; **54**: 273-80.
142. Wang X. Effects of different intercropping models on uptake and runoff loss of nutrient, crop yield and economic benefits. Hunan Agricultural University, Hunan Agricultural University, 2015.
143. Wang X. Effect of biochar addition on greenhouse gas emissions and nitrogen loss from tea planted soil. Zhejiang University, Zhejiang University, 2017.
144. Wang X. Effects of nitrogen fertilizer types on nitrogen and carbon emissions and non-point source pollution in saline-alkali paddy fields and environmental risk assessment, Northeast Institute of Geography and Agroecology, Chinese Academy of Sciences 2024.
145. Wang X, Gao R, Qian X *et al.* Nitrogen loss via runoff from paddy field using the large catchment area in taihu region. *Journal of Agro-Environment Science* 2007: 831-5.
146. Wang X, Wang Y, Tian X *et al.* Effects of NMUrea on nitrogen runoff losses of surface water and nitrogen fertilizer efficiency in paddy feild. *Transactions of the Chinese Society of Agricultural Engineering* 2011; **27**: 106-11.
147. Wang X, Wang Y, Wang X *et al.* The character of nutrient loss and land use in a small watershed of Miyun Reservoir. *research of environmental sciences* 2003: 30-3.
148. Wang X, Zhu J, Gao R *et al.* Nitrogen cycling and losses under rice-wheat rotations with coated urea and urea in the Taihu Lake Region. *Pedosphere* 2007; **17**: 62-9.
149. Wang X, Gao R, Zhu J *et al.* Nitrogen loss via runoff and leaching from employ of different urea bleeds in paddy season. *China Environmental Science* 2004: 89-93.
150. Wang Y, Han X, Zhao Z *et al.* Controlling effects of furrow straw mulching on N and P nutrient loss on black soil slope farmland. *Journal of Soil and Water Conservation* 2016; **30**: 137-40.
151. Wang Y, Qiu X, Hu G *et al.* Effect of controlled release fertilizer on nitrogen and phosphorus runoff losses from farmland in slope field. *Journal of Soil and Water Conservation* 2011; **25**: 10-4.
152. Wen Y, Zhang H, Xie J *et al.* Studies on the nitrogen loss from rice field with different fertilizer treatments. *Bulletin of Science and Technology* 2011; **27**: 549-53.
153. Wu J, Fan J, He Y *et al.* Dynamics of nitrogen and runoff loss in ponding water of paddy field under different fertilization practices. *Ecology and Environmental Sciences* 2012; **21**: 1561-6.
154. Wu X, Chen S. Study on the nitrogen and phosphrous losses pollution from nonirrigated lateritic red earth. *Journal of Zhanjiang Ocean University* 1999: 58-62.
155. Xi Y, Wang H, Xu X *et al.* Research on the patterns and control measures of nitrogen and phosphorus runoff loss during the rice growing season in the Taihu Lake Basin. In: *The 2009 China Environmental Science Society Academic Annual Conference,* *2009*, p. 7.
156. Xia S, Qiao Y, Zhu J. Reducing nitrogen loss from paddy filed and ensuring yield of direct seeding rice by adjusting nitrogen base-topdressing ratio. *Acta Agriculturae Zhejiangensis* 2022; **34**: 2482-90.
157. Xia Y, Wang R, Gao L *et al.* Runoff losses characteristics of nitrogen and phosphorus from rapeseed-cotton rotation systems in Hubei Province. *Hubei Agricultural Sciences* 2014; **53**: 5751-4.
158. Xiao Q, Zhang W, Wang Q *et al.* Nitrogen losses under simulated rainfall conditions in rice-wheat rotation of Taihu lake watershed plain. *Plant Nutrition & Fertilizing Science* 2005: 25-30.
159. Xie R, Tan H, Zhou L *et al.* Water, soil, nitrogen and phosphorus losses from surface runoff in sugarcane cropping system. *Southwest China Journal of Agricultural Sciences* 2013; **26**: 1572-7.
160. Xie X, Chen J, Tang L *et al.* Effects of different p fertilizer application rates on soil nitrogen and phosphorus losses with surface runoff during rice season. *Acta Agriculturae Boreali-Occidentalis Sinica* 2007: 261-6.
161. Xu J, Shan L, Yu D *et al.* Effects of different fertilization modes on nitrogen use efficiency of cabbages and nitrogen loss from vegetable field. *Journal of Zhejiang University:Agriculture and Life Sciences* 2013; **39**: 556-64.
162. Xu J, Sui S, Kang Y *et al.* Characteristics of nitrogen and phosphorus loss in dry farmland of Liaohe River Plain. *Journal of Maize Sciences* 2021; **29**: 131-9.
163. Xu Y, Wu L, Wu W *et al.* Effects of nitrogen application on wheat yield and runoff loss of nitrogen and application threshold of nitrogen fertilizer. *Journal of Soil and Water Conservation* 2018; **32**: 246-51.
164. Xue L, Yu Y, Yang L. Nitrogen balance and environmental impact of paddy field under different N management methods in Taihu Lake region. *Environmental Science* 2011; **32**: 1133-8.
165. Xue L, Yu Y, Yang L. Maintaining yields and reducing nitrogen loss in rice–wheat rotation system in Taihu Lake region with proper fertilizer management. *Environmental Research Letters* 2014; **9**.
166. Yan J, He B, Tian T. Effect of fertilizer levels and tillage methods on soil erosion and nutrient loss in purple soil area. *Scientia Agricultura Sinica* 2014; **47**: 4027-35.
167. Yan K, Ma M, Li W *et al.* Effects of different fertilization patterns on nitrogen and phosphorus loss in the operatic circle in coastal areas of Jiangsu. *Journal of Zhejiang Agricultural Sciences* 2023; **64**: 2181-7.
168. Yan L. Study on agricultural non-point pollution impact of different fertilization management in maize continuous cropping area in Northeast China. Jilin University, Jilin University, 2016.
169. Yan L, Cao M, Hu Z *et al.* The effect of fertilization method on nitrogen and phosphorus loss in maize planting zone in Northeast China. *Environmental Pollution & Control* 2018; **40**: 170-175+180.
170. Yan L, Feng G, Lu W *et al.* The effect of fertilization method on nitrogen and phosphorus loss by runoff and leaching in dry years. *China Rural Water and Hydropower* 2017: 32-5.
171. Yan L, Gao Q, Zhang Z *et al.* Effect of reducing fertilizer and resource recycling on nitrogen and phosphorus loss on black soil with planting maize in Northeast region under natural rainfall condition. *Journal of Soil and Water Conservation* 2014; **28**: 1-6+103.
172. Yan L, Wang Y, Feng G *et al.* Effect of different fertilization management on nitrogen loss in black soils in Northeast China. *Journal of Agro-Environment Science* 2016; **35**: 1816-23.
173. Yan W. Effect of slow-controlled release fertilizer and reducing nitrogen application on loss and utilization of nitrogen and upland crop yield. Hunan Agricultural University, Hunan Agricultural University, 2014.
174. Yang S, Peng S, Xu J *et al.* Effects of water saving irrigation and controlled release nitrogen fertilizer managements on nitrogen losses from paddy fields. *Paddy and water environment* 2015; **13**: 71-80.
175. Yang X, Zhang Q, Lu X *et al.* Effects of controlled-release compound fertilizer on peanut yield and nitrogen loss in sloping farmland. *Journal of Plant Nutrition and Fertilizers* 2023; **29**: 1751-60.
176. Yang Y. Effects of specific fertilizer on runoff loss of nitrogen and phosphorus and yield of rice. *Hunan Agricultural Sciences* 2011: 42-4.
177. Yang S, Xing L, Liu H *et al.* Effect of planting patterns on soil nitrogen and phosphorus loss in slope farmland in the main Songhua river basin. *Journal of Northwest A & F University(Natural Science Edition)* 2018; **46**: 61-9.
178. Yang Y. Effects of two carbon sources on nitrogen budget of vegetablered soil. Yunnan Agricultural University, Yunnan Agricultural University, 2022.
179. Yang Y, Li C, Peng Y *et al.* Effects of biochar and organic fertilizer application on physical and chemical properties of red soil and nitrogen losses by runoff. *Soil and Fertilizer Sciences in China* 2023: 16-24.
180. Yao J, Ning J, Li M *et al.* Characteristics of nitrogen runoff from paddy fields in Guangdong Province. *Journal of Agro-Environment Science* 2015; **34**: 728-37.
181. Yao J, Tang C, He B. Effects of different fertilization level on N-losses along with surface runoff on slope land of purple soil. *Research of Soil and Water Conservation* 2010; **17**: 54-7.
182. Ye Z, Liao M, Huang Y *et al.* Influence of different management mode of fertilization on nitrogen losses in runoff from nursery land in a catchment area. *Journal of Soil and Water Conservation* 2016; **30**: 30-7.
183. Yi X. Features associated with nitrogen loss and microbial communities in rice-frog cultivation. Shanghai Jiao Tong University, Shanghai Jiao Tong University, 2020.
184. Yu J, Zhao X, Lu B *et al.* Transportation characteristics of nitrogen and phosphorus in various land-uses in the Poyan Lake watershed. *Acta Agriculturae Universitatis Jiangxiensis* 2010; **32**: 394-402.
185. Yu P. Characteristic of nitrogen loss from farmland in guangdong west by natural rainfall runoff. South China University of Technology, South China University of Technology, 2011.
186. Yu Q, Ye J, Ma J *et al.* Effects of different nitrogen applied levels on nitrogen runoff loss in oilseed rape fields. *Journal of Soil & Water Conservation* 2011; **25**: 22-25+30.
187. Yu Y, Xue L, Yang L. Nitrogen use efficiency and loss from runoff and leaching in wheat season with rice-wheat rotation system under different nitrogen management methods in Taihu Lake Region, China. *Journal of Agro-Environment Science* 2011; **30**: 2475-82.
188. Yuan D, Wang Z, Chen X *et al.* Characteristics of nitrogen loss from sloping field in red soil area under different cultivation practices. *Chinese Journal of Applied Ecology* 2002; **13**: 863-6.
189. Yuan H, Huang S, Kong X *et al.* Effects of different fertilization models on runoff loss of nitrogen and phosphorus in early rice paddy fields. *Research of Agricultural Modernization* 2021; **42**: 776-84.
190. Zeng Z, Ai S, Yao J *et al.* Effect of fertilization on nitrogen runoff from early paddy field in the Pearl River Delta Region. *Guangdong Agricultural Sciences* 2010; **37**: 27-30.
191. Zhang B. Effects of stand transformation of eucalyptus forest on soil nutrient and nutrient loss via surface runoff. South China Agricultural University, South China Agricultural University, 2016.
192. Zhang D, He R, Zheng J *et al.* Investigation on water quality and runoff loss of nitrogen and phosphorus from the typical agricultural watersheds in three gorges reservoir area — a case study in Lianfeng, Fuling District. *Environment and Ecology in the Three Gorges* 2011; **33**: 1-5.
193. Zhang K. The effects of rotation modes and fertilizer types on the loss of rice track and field flow and nutrient utilization. Zhejiang A&F University, Zhejiang A&F University, 2021.
194. Zhang L, Wu X, Song P *et al.* Study on resistance and control technology of nitrogen and phosphorus nutrient loss in freeze‐thaw farmland. *Journal of Henan Agricultural Sciences* 2023; **52**: 88-96.
195. Zhang M, Wang Y, Huang C. Characteristics of nitrogen and phosphorus runoff losses from croplands with different planting patterns in a riverine plain area of Zhejiang Province, East China. *Chinese Journal of Applied Ecology* 2011; **22**: 3211-20.
196. Zhang M, Zhao M, Tian Y *et al.* Study on N leaching and runoff under integrated high yield and high efficiency practices in paddy fields of Taihu Lake Region. *Soils* 2018; **50**: 35-42.
197. Zhang X. Soil nitrogen loss in runoff and sediment as affected by level trench and crop rotation. *Transactions of the Chinese Society of Agricultural Engineering* 2002: 70-73.
198. Zhang Y, Ding H, Zheng X *et al.* Nitrogen and phosphorus losses from surface runoff in tobacco-rice rotation system in northwest of Fujian Province, China. *Journal of Agro-Environment Science* 2012; **31**: 969-76.
199. Zhang Y, Rong X, Liu Q *et al.* Effects of combination of organic and inorganic fertilizer on crop nutrient utilization rate, nitrogen and phosphorus loss in dry land. *Journal of Soil and Water Conservation* 2013; **27**: 44-48+54.
200. Zhang Y, Rong X, Wang X *et al.* Effects of ecology interception and film mulching on surface runoff and nitrogen loss in upland soil. *Journal of Soil and Water Conservation* 2014; **28**: 15-19. doi: 10.13870/j.cnki.stbcxb.2014.02.003
201. Zhang Z, Dong L, Zhu Y. The dynamic characteristics and modeling of nitrogen in paddy field surface water and nitrogen loss from field drainage. *Acta Scientiae Circumstantiae* 2001: 475-80.
202. Zhao D, Yan T, Qiao J *et al.* Characteristics of N loss and environmental effect of paddy field in Taihu area. *Ecology and Environmental Sciences* 2012; **21**: 1149-1154. doi: 10.16258/j.cnki.1674-5906.2012.06.016
203. Zhao H, Liu G, Cao Q *et al.* Influence of different land use types on soil erosion and nutrition care effect in loess hilly region. *Journal of Soil and Water Conservation* 2006: 20-24+54.
204. Zhao X, Xie Y, Xiong Z *et al.* Nitrogen fate and environmental consequence in paddy soil under rice-wheat rotation in the Taihu lake region, China. *Plant and Soil* 2009; **319**: 225-34.
205. Zheng W, Tan W, Liu Z *et al.* Effect of reduced nitrogen fertilization on nitrogen runoff loss and apparent balance on red-soil dry slope peanut land. *Acta Pedologica Sinica* 2022; **59**: 1540-50.
206. Zhou Q, Zhu J, Zou Y *et al.* Effects of polymer-coated urea combined with common urea on ammonia volatilization,nitrogen runoff and leakage loss,and yield in direct-seeding paddy field. *Journal of Agro-Environment Science* 2023; **42**: 2016-26.
207. Zhou W, Sun G, Wang X *et al.* Risk of nitrogen loss under the combined application of biogas slurry and organic fertilizer. *Journal of Agro-Environment Science* 2019; **38**: 1743-50.
208. Zhu J, Gao C, Zhu J *et al.* Nitrogen exports via overland runoff under different land uses and their seasonal pattern. *Journal of Nanjing University(Natural Sciences)* 2006: 621-7.
209. Zhu W, Liu M, Xiao X *et al.* Effects of chemical fertilizer combined with organic manure on early rice yield and nitrogen fate in paddy field. *Journal of Shenyang Agricultural University* 2019; **50**: 728-33.
210. Zhu X, Sheng H, Xia X *et al.* Nitrogen loss with runoff and drainage from farmland under wheat-rice rotation. *Journal of Ecology and Rural Environment* 2006: 38-41+66.
211. Zhao Z, Cao L, Sha Z *et al.* Impacts of fertilization optimization on N loss from paddy fields: Observations and DNDC modeling case study in Shanghai, China. *Soil and Tillage Research* 2020; **199**: 104587.
212. Bian L, Jiao J, Li H *et al.* Modeling of nitrogen loss with runoff from paddy field during rice growth period in Nanjing area. *Journal of Ecology and Rural Environment* 2008; **24**: 89-93.
213. Li X, Zhang W, Wu J *et al.* Loss of nitrogen and phosphorus from farmland runoff and the interception effect of an ecological drainage ditch in the North China Plain—A field study in a modern agricultural park. *Ecological Engineering* 2021; **169**: 106310.
214. Jiao S, Hu X, Pan G *et al.* Effects of fertilization on nitrogen and phosphorus run-off loss from Qingzini paddy soil in Taihu Lake region during rice growth season. *Chinese Journal of Ecology* 2007; **26**: 495.
215. You L, Ros GH, Chen Y *et al.* Optimized agricultural management reduces global cropland nitrogen losses to air and water. *Nature Food* 2024; **5**: 995-1004.
216. Xue L, Hou P, Zhang Z *et al.* Application of systematic strategy for agricultural non-point source pollution control in Yangtze River basin, China. *Agriculture, Ecosystems & Environment* 2020; **304**: 107148.
217. Xue L, Yu Y, Yang L. Maintaining yields and reducing nitrogen loss in rice–wheat rotation system in Taihu Lake region with proper fertilizer management. *Environmental Research Letters* 2014; **9**: 115010.
218. Gu B, Zhang X, Lam SK *et al.* Cost-effective mitigation of nitrogen pollution from global croplands. *Nature* 2023; **613**: 77-84.
219. Ding W, He P, Zhang J *et al.* Optimizing rates and sources of nutrient input to mitigate nitrogen, phosphorus, and carbon losses from rice paddies. *Journal of Cleaner Production* 2020; **256**: 120603.
220. Hou P, Jiang Y, Yan L *et al.* Effect of fertilization on nitrogen losses through surface runoffs in Chinese farmlands: A meta-analysis. *Science of The Total Environment* 2021; **793**: 148554.
221. Li P, Lu J, Wang Y *et al.* Nitrogen losses, use efficiency, and productivity of early rice under controlled-release urea. *Agriculture, Ecosystems & Environment* 2018; **251**: 78-87.
222. Su N, Ronga X, Xie G *et al.* Effectiveness of a 10-year continuous reduction of controlled-release nitrogen fertilizer on production, nitrogen loss and utilization of double-cropping rice. *Science of The Total Environment* 2024; **912**: 168857.
223. Xia L, Lam SK, Chen D *et al.* Can knowledge‐based N management produce more staple grain with lower greenhouse gas emission and reactive nitrogen pollution? A meta‐analysis. *Global Change Biology* 2016; **23**: 1917-25.
224. Shan L, He Y, Chen J *et al.* Nitrogen surface runoff losses from a Chinese cabbage field under different nitrogen treatments in the Taihu Lake Basin, China. *Agricultural Water Management* 2015; **159**: 255-63.
225. Ren J, Hu K, Feng P *et al.* Simulating Responses of Rice Yield and Nitrogen Fates to Ground Cover Rice Production System under Different Types of Precipitation Years. *Rice Science* 2024; **31**: 725-39.
226. Wang J, Lü G, Guo X *et al.* Conservation tillage and optimized fertilization reduce winter runoff losses of nitrogen and phosphorus from farmland in the Chaohu Lake region, China. *Nutrient Cycling in Agroecosystems* 2015; **101**: 93-106.
227. Tao W, Li J, Li W *et al.* Higher rice productivity and lower paddy nitrogen loss with optimized irrigation and fertilization practices in a rice-upland system. *Agriculture, Ecosystems & Environment* 2024; **374**: 109176.
228. Han Y, Liu Z, Chen Y *et al.* Assessing non-point source pollution in an apple-dominant basin and associated best fertilizer management based on SWAT modeling. *International Soil and Water Conservation Research* 2023; **11**: 353-64.
229. Yang G, Best EP. Spatial optimization of watershed management practices for nitrogen load reduction using a modeling-optimization framework. *Journal of Environmental Management* 2015; **161**: 252-60.
230. Fan H, Huang Z, Feng C *et al.* Functional keystone taxa promote N and P removal of the constructed wetland to mitigate agricultural nonpoint source pollution. *Science of The Total Environment* 2024; **912**: 169155.
231. Lyu C, Li X, Yuan P *et al.* Nitrogen retention effect of riparian zones in agricultural areas: A meta-analysis. *Journal of Cleaner Production* 2021; **315**: 128143.
232. Xie W, Li J, Liu Y *et al.* Evaluation of ecological buffer zone based on landscape pattern for non-point source pollution control: A case study in Hanjiang River basin, China. *Journal of Hydrology* 2023; **626**: 130341.
233. Zhang B, Chen Y, Chen X *et al.* Effectiveness and resilience of BMPs to watershed climate adaptation considering the uncertainty of hydrological model and GCMs. *Climate Risk Management* 2024; **44**: 100612.
234. Tang W, Zhang W, Zhao Y *et al.* Nitrogen removal from polluted river water in a novel ditch–wetland–pond system. *Ecological Engineering* 2013; **60**: 135-9.
235. Bai J, Qin Y, Zhao J *et al.* Investigation of agricultural nutrient removal by ecological ditches using meta-analysis. *Agriculture, Ecosystems & Environment* 2025; **380**: 109401.
236. Shen W, Li S, Mi M *et al.* What makes ditches and ponds more efficient in nitrogen control? *Agriculture, Ecosystems & Environment* 2021; **314**: 107409.
237. Yan X, Han H, Li X *et al.* Dissolved organic carbon and dissolved oxygen determine the nitrogen removal rate constant in small water bodies of intensive agricultural region. *Agriculture, Ecosystems & Environment* 2024; **361**: 108822.
238. Zhao Y, Yang Z, Xia X *et al.* A shallow lake remediation regime with Phragmites australis: Incorporating nutrient removal and water evapotranspiration. *Water Research* 2012; **46**: 5635-5644.
239. Cui Z, Huang J, Gao J *et al.* Characterizing the impacts of macrophyte-dominated ponds on nitrogen sources and sinks by coupling multiscale models. *Science of The Total Environment* 2022; **811**: 152208.
240. Zhang S-Y, Liu X, Hao B *et al.* Nitrogen removal performance and mechanisms of three aquatic plants for farmland tail water purification. *Science of The Total Environment* 2024; **917**: 170524.
241. Shen Y-T, Hou S-N, Hu S-L *et al.* Water purification capacity of ecological ditch: A systematic review and meta-analysis of influencing factors. *Ecological Engineering* 2024; **204**: 107280.
242. Ge Z, Feng C, Wang X *et al.* Seasonal applicability of three vegetation constructed floating treatment wetlands for nutrient removal and harvesting strategy in urban stormwater retention ponds. *International Biodeterioration & Biodegradation* 2016; **112**: 80-7.
243. Wu J, Cao X, Zhao J *et al.* Performance of biofilter with a saturated zone for urban stormwater runoff pollution control: Influence of vegetation type and saturation time. *Ecological Engineering* 2017; **105**: 355-61.
244. Zhang W, Li H, Pueppke SG. Direct measurements of dissolved N2 and N2O highlight the strong nitrogen (N) removal potential of riverine wetlands in a headwater stream. *Science of The Total Environment* 2022; **848**: 157538.
245. Yin X, Jiang C, Zheng X *et al.* Integrated constructed wetland-river (ICWR) system for efficient nitrogen removal from secondary effluent: Performance, cost and carbon footprint analysis. *Journal of Environmental Management* 2025; **388**: 125984.
246. Du L, Trinh X, Chen Q *et al.* Enhancement of microbial nitrogen removal pathway by vegetation in Integrated Vertical-Flow Constructed Wetlands (IVCWs) for treating reclaimed water. *Bioresource Technology* 2018; **249**: 644-51.
247. Zhang S, Xiao R, Liu F *et al.* Effect of vegetation on nitrogen removal and ammonia volatilization from wetland microcosms. *Ecological Engineering* 2016; **97**: 363-9.
248. Wang J, Chen G, Fu Z *et al.* Application performance and nutrient stoichiometric variation of ecological ditch systems in treating non-point source pollutants from paddy fields. *Agriculture, Ecosystems & Environment* 2020; **299**: 106989.
249. Shen W, Zhang L, Ury EA *et al.* Restoring small water bodies to improve lake and river water quality in China. *Nature Communications* 2025; **16**: 294.
250. Zhang W, Li H, Kendall AD *et al.* Nitrogen transport and retention in a headwater catchment with dense distributions of lowland ponds. *Science of The Total Environment* 2019; **683**: 37-48.
251. Wang Z, Dong Y, Xu J *et al.* Nitrogen removal performance and microbial community characteristics of a submerged macrophyte pond–constructed wetland composite system. *Journal of Water Process Engineering* 2025; **72**: 107492.
252. Lv M, Chen J, Ma M *et al.* Diminished storage capacity of ponds caused by sedimentation weakens their nitrogen removal efficiency. *Water Research* 2024; **261**: 121987.
253. Li S, Liu H, Zhang L *et al.* Potential nutrient removal function of naturally existed ditches and ponds in paddy regions: Prospect of enhancing water quality by irrigation and drainage management. *Science of The Total Environment* 2020; **718**: 137418.
254. Li D, Chu Z, Huang M *et al.* Multiphasic assessment of effects of design configuration on nutrient removal in storing multiple-pond constructed wetlands. *Bioresource Technology* 2019; **290**: 121748.
255. Wang T, Xiao L, Lu H *et al.* Nitrogen removal from summer to winter in a field pilot-scale multistage constructed wetland-pond system. *Journal of Environmental Sciences* 2022; **111**: 249-62.
256. Li Y, Zhang H, Zhu L *et al.* Evaluation of the long-term performance in a large-scale integrated surface flow constructed wetland–pond system: A case study. *Bioresource Technology* 2020; **309**: 123310.
257. Beusen AH, Bouwman AF, Van Beek LP *et al.* Global riverine N and P transport to ocean increased during the 20th century despite increased retention along the aquatic continuum. *Biogeosciences* 2016; **13**: 2441-51.
258. Li D, Chu Z, Li P *et al.* Impacts of landscape spatial configuration of integrated multi-pond constructed wetlands in a basin on the treatment of non-point source pollution. *Journal of Cleaner Production* 2023; **383**: 135389.
259. Sun C, Chen L, Liu H *et al.* New modeling framework for describing the pollutant transport and removal of ditch‐pond system in an agricultural catchment. *Water Resources Research* 2021; **57**: e2021WR031077.
